# A synthetic population-level oscillator in non-microfluidic environments

**DOI:** 10.1038/s42003-023-04904-0

**Published:** 2023-05-13

**Authors:** Fei Gu, Wei Jiang, Fangbing Kang, Tianyuan Su, Xiaoya Yang, Qingsheng Qi, Quanfeng Liang

**Affiliations:** 1 State Key Laboratory of Microbial Technology, Shandong University, No. 72, Binhai Road, 266237 Qingdao, China; 2 Research Center of Basic Medicine, Central Hospital Affiliated to Shandong First Medical University, Jinan, China

**Keywords:** Genetic circuit engineering, Oscillators, Metabolic engineering

## Abstract

Synthetic oscillators have become a research hotspot because of their complexity and importance. The construction and stable operation of oscillators in large-scale environments are important and challenging. Here, we introduce a synthetic population-level oscillator in *Escherichia coli* that operates stably during continuous culture in non-microfluidic environments without the addition of inducers or frequent dilution. Specifically, quorum-sensing components and protease regulating elements are employed, which form delayed negative feedback to trigger oscillation and accomplish the reset of signals through transcriptional and post-translational regulation. We test the circuit in devices with 1 mL, 50 mL, 400 mL of medium, and demonstrate that the circuit could maintain stable population-level oscillations. Finally, we explore potential applications of the circuit in regulating cellular morphology and metabolism. Our work contributes to the design and testing of synthetic biological clocks that function in large populations.

## Abstract (web summary)

A synthetic population-level oscillator in *Escherichia coli* is designed in non-microfluidic environments without the addition of inducers or frequent dilution.

## Introduction

Circadian clocks are ubiquitous in life on the earth, allowing organisms to anticipate environmental changes and complete normal physiological processes such as metabolism and circadian rhythms^1^. The circadian clock relies on single-cell oscillations and synchronization between oscillations. The periodic increase and decrease of mRNA and protein in cells are usually regulated by transcription or translation feedback loops, known as genetic oscillators^2^. In natural genetic oscillators, cells and tissues achieve periodic behavior through biochemical networks that contain multiple regulatory feedback loops, such as the core clock network of eukaryotes consisting of dozens of genes^3,4^. The complexity of natural clocks hinders the deconstruction, analysis and application of oscillators. Synthetic genetic networks provide a relatively controlled test platform, in which components of natural networks are separated and analyzed in detail, and then cleverly assembled into engineered artificial networks to perform predictable functions^5^. Synthetic oscillators could provide a controlled, simplified and orthogonal system for investigating the core genetic structures of the circadian clock, which is not only important for understanding the underlying mechanisms of the circadian clock, but also has many potential applications in fields such as bioengineering and biomedicine^6,7^.

In synthetic oscillation circuits, negative feedback loops can trigger oscillations^8,9^. Goodwin oscillator is the simplest model of limit cycle oscillations caused by one negative feedback, inspiring a series of subsequent studies^10–12^. Repressilator consists of three blocks of genes that are repressed in turn, causing significant oscillations in individual cells of *Escherichia coli* (*E. coli*)^13^. Computational studies have shown that positive feedback loops endow the synthetic oscillator with tunability and robustness^14,15^. The dual-feedback oscillator exhibits excellent robustness and tunability under microfluidic platform^16^. Most biological clocks rely on transcriptional-translational negative feedback loops, but also on post-translational regulation^17,18^. Multi-level regulation might provide more possibilities for the optimization and upgrading of genetic circuits. Fernandez-Rodriguez and Voigt demonstrated that the three Potyvirus proteases, TEV protease (TEVp), TVMV protease (TVMVp), and SUMMV protease (SUMMVp), were highly orthogonal, and showed that the domains could be shuffled to modify the response of a synthetic circuit and increase the dynamic range of output^19^. Controllable proteolysis offers a powerful tool for modulating and expanding the function of synthetic circuits, such as negative and positive feedback at the post-translation level^20–22^.

Synthetic oscillators synchronizing at the population level are more similar to naturally evolved circadian clock circuits. Currently, the synchronization of synthetic oscillators is achieved mainly by eliminating noise and coupling^23,24^. Biochemical noise is one of the challenges to the stable operation of synthetic circuits, a single stochastic signal step can introduce fundamental constraints that cannot be overcome by any control system^25^. The synchronization of the Repressilator without coupling can be achieved by reducing error propagation and information loss^26,27^. Recently, a robust CRISPR-interference-based Repressilator, the CRISPRlator, allows for synchronous three-color oscillations by reducing noise variation^28^. The synchronization by reducing noise is implemented in a manner similar to that of cyanobacterial clocks. However, most biological clocks of plants or animals synchronize multiple oscillators through coupling to resist environmental disturbances and maintain oscillations flexibly^29,30^. Quorum sensing (QS) can synchronize the behavior of bacteria in response to population density, which provides an important means to achieve synchronization of synthetic oscillators^6,31^. The most widely studied oscillator coupled by QS is the synchronized genetic clock, which is coupled by Lux QS, showing stable population-level oscillations on the microfluidic platform^32^. Din et al. further constructed a synchronized lysis circuit using bacteriophage lysis gene as a negative feedback element in the basis of synchronized genetic clock, achieving periodic population lysis events under microfluidic platform^33^.

At present, synthetic oscillators are mostly tested on microfluidic platforms, which provide intuitive support for the improvement of the circuits. However, there are some limitations. The fluid volume is small, usually less than one microliter, making it difficult to collect samples for downstream analysis. Furthermore, microfluidic chambers are relatively small and will be affected by laminar flow, while biological behavior in nature develops macroscopic structures and will experience the effects of turbulence^26,34–36^. Additional operations and further improvements are often required to test and apply synthetic oscillators in non-microfluidic environments^37^. An optimized Repressilator and a protease-based oscillator could perform oscillation behavior in the flasks, but only following the addition of inducers to synchronize bacteria and dilution every 50 min or two hours to maintain cell activity^22,26^. The engineered bacteria containing the synchronized lysis circuit could show “increase-decrease-increase” pulsatile population dynamics in vivo, demonstrating the potential of synthetic oscillators for biotherapy and drug delivery^33^. To construct continuous synthetic population-level oscillators in non-microfluidic environment and to explore their application potential in different fields are challenging and necessary^38^.

Here, we constructed a continuous synthetic population-level oscillator in non-microfluidic environments without the addition of inducers or frequent dilution operations. The circuit was designed with QS components and protease regulatory elements to trigger oscillation and complete the propagation and multiple resets of QS signals. We tested the circuit in 24-well plates, shake flasks and quadruple tanks, and explored the potential applications of the oscillator in regulating cellular morphology and metabolism. Our work provides useful information for the design, testing and application of synthetic clocks in non-microfluidic environments.

## Results

### The initial design version of the QS-protease oscillator

Regarding QS coupling oscillators, the most important one is the synchronized genetic clock, which exhibits regular and robust population-level oscillations on the microfluidic platform. In this system, LuxI synthase produces the signal molecule acyl homoserine lactone (AHL), which diffuses and mediates intercellular coupling. AHL binds to LuxR and then activates the expression of LuxI and AiiA controlled by the promoter P_lux_, while AiiA degrades AHL to crate negative feedback^32^. We constructed the circuit according to the above mechanism, and tested it in a 24-well plate with 1 mL of medium. The overall fluorescence of the circuit fluctuated, but the oscillation behavior was not regular and not obvious after OD standardization (Supplementary Fig. 1). We speculated that the reason why the circuit could not oscillate continuously in the 24-well plate might be related to the continuous completion of signal reset under the condition of large-scale culture. The initiation of this circuit is caused by the leakage of LuxI to generate AHL. However, the leakage expression of LuxI with the degradation tag is relatively weak, and thus it is slow to restart the circuit in the stationary phase and on a large-scale culture. In addition, both the synthesis component LuxI and the degradation component AiiA are controlled by the same promoter P_lux_, suggesting that the AHL synthesis and degradation processes are to some extent hedging, which may have an impact on the reset of QS signals under specific culture conditions.

**Fig. 1:**
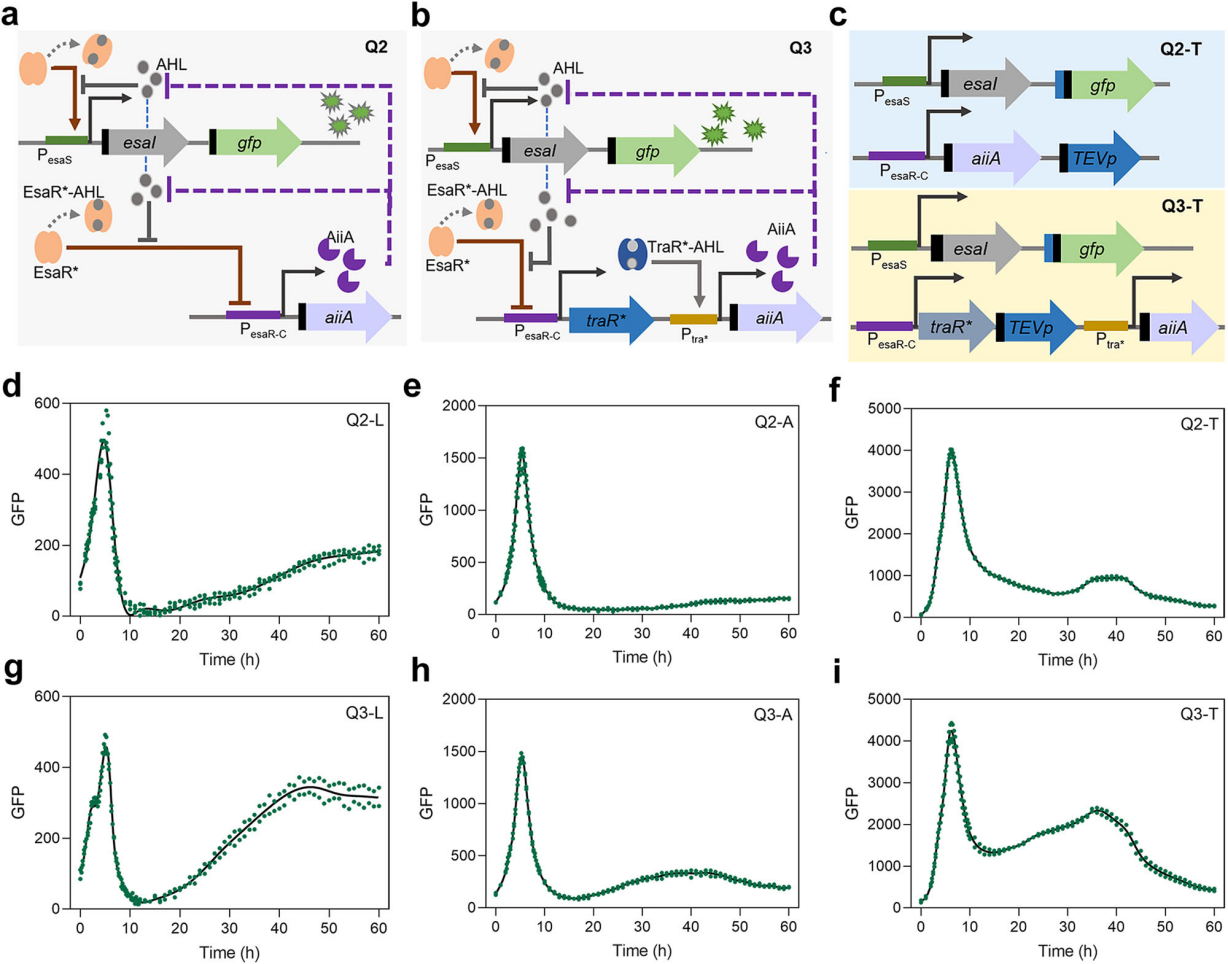
Design and characterization of Q2 and Q3. **a** Q2 circuit regulated by Esa QS-switch. Initially, EsaR* activates the expression of EsaI and GFP controlled by P_esaS_ and represses the expression of AiiA controlled by P_esaR-C_. When AHL signals accumulate to a certain extent, the expression of EsaI and GFP would be shut down, AHL is not produced, and AiiA controlled by P_esaR-C_ begins to express. Then, AiiA degrades AHL to accelerate signal reset. The black figure before the gene module in the diagrams represents the degradation tag. **b** Q3 circuit regulated by Esa QS-switch and cascade components. Initially, EsaR* activates the expression of EsaI and GFP controlled by P_esaS_. When AHL signals accumulate to a certain extent, the expression of EsaI and GFP would be shut down, AHL is not produced, and TraR* controlled by P_esaR-C_ begins to express. Then, TraR* binds to AHL to activate the expression of AiiA controlled by P_tra*_, and AiiA degrades AHL to accelerate signal reset. The black figure in the diagrams represents the degradation tag. **c** The circuit composition of Q2-T and Q3-T. GFP exists stably in the absence of protease, and when the TEV protease is exist, TEV cut off the corresponding cleavage tag, exposing the proteolytic tag and leading to rapid degradation of GFP. The black figure next to the *gfp* figure represents the LVA tag and the dark blue figure represents the TEV protease cleavage tag. **d**–**i** The overall fluorescence characterization of Q2-L, Q2-A, Q2-T, Q3-L, Q3-A, Q3-T in 24-well plates. The green dots are the measuring results of two groups of independent samples. The black solid line is the fitting curve generated by GraphPad Prism software.

From the point of view of signal reset, separating the processes of signal synthesis and degradation may be helpful for oscillations in non-microfluidic environments. Our previously published dual-function QS-switch based on Esa QS might circumvent this problem. In Esa QS, EsaR can function as a transcriptional activator of the promoter P_esaS_ and a transcriptional repressor of the promoter P_esaR_. When AHL reaches a certain threshold, it binds to EsaR to detach it from DNA, and the expression of genes controlled by P_esaS_ is dynamically downregulated, while the expression of genes controlled by P_esaR_ is dynamically upregulated. P_esaS_ and P_esaR-C_ are able to simultaneously up-regulate and down-regulate different genes in the regulation process of Esa QS^39–42^. We designed an oscillator driven by the Esa QS-switch, named Q2. In circuit Q2, the expression of AHL synthase EsaI and reporter green fluorescent protein (GFP) is controlled by P_esaS_, and the expression of AHL degrading enzyme AiiA is controlled by P_esaR-C_. While EsaRI70V (EsaR*) activates the expression of EsaI and GFP, it inhibits the expression of AiiA. When AHL accumulates to a certain threshold, it binds to EsaR*, thereby turning off the expression of EsaI and GFP, while turning on the expression of AiiA, which degrades AHL until the signal is reset and the next cycle is initiated (Fig. 1a). The dual-function QS-switch separates the synthesis and degradation of AHL in Q2. In this process, the accumulated AHL binds to EsaR* and turns off the expression of genes controlled by P_esaS_, forming the delayed negative feedback at the population level to drive the emergence of oscillation. The mechanism is similar to the coupled Goodwin oscillator^10–12,43,44^. The accumulated AHL could aslo turn on AiiA expression to degrade AHL to prevent signal overload. The activation element is designed as a measure to reinforce negative feedback, similar to the effect of positive feedback in some oscillation circuits^45–47^. In addition, inspired by the synthetic cascade element constructed by colleagues in our laboratory^48^, we tried to optimize Q2 to further increase the delay of enhanced negative feedback, and constructed the oscillator Q3. In circuit Q3, P_esaR-C_ controls the expression of TraR variant (TraR*)^48^, and the binding of TraR* to AHL activates the expression of AiiA controlled by P_tra*_ (Fig. 1b).

To ensure that GFP could reflect the changes of the circuit more accurately, we used the stronger degradation tag LVA, the weaker degradation tag AAV and the LVA-TEV tag regulated by TEVp to regulate the turnover rate of GFP^19,22^ (Fig. 1c). The combinations of this part were designated Q2-L, Q2-A, Q2-T, Q3-L, Q3-A and Q3-T. In Q2-L and Q3-L, Q2-A and Q3-A, and Q2-T and Q3-T, the degradation tags of GFP were LVA, AAV and LVA-TEV tags, respectively. The above combinations were tested in 24-well plates with 1 mL of medium. From the overall fluorescence of bacteria, all circuits were observed to be fluctuating. Both Q3 and Q2 oscillation circuits could achieve two oscillations, although the second oscillation was not obvious, with a long period and low amplitude (Fig. 1d–i). Compared with Q2, the oscillation of Q3 was more obvious. This may be due to the delayed enhanced negative feedback, or the fact that additional TraR* binding to AHL reduces the concentration of AHL to some extent and activates the expression of AiiA to degrade AHL to achieve signal reset as soon as possible. Q2-T and Q3-T showed more pronounced oscillations than the other combinations, possibly because the additional post-translational regulation slowed the effect of the strong degradation tag, making fluorescence changes during the second oscillation more pronounced. However, for the fluorescence characterization results after OD standardization, the first oscillation of all combinations was obvious, but the subsequent oscillation was weak or almost invisible (Supplementary Fig. 2). The above results indicated that the circuit Q3-T combining transcriptional regulation and post-translational regulation exhibited significant fluctuations, but further optimization was needed to achieve more consummate oscillations.

### The optimized design version of the QS-protease oscillator

**Fig. 2:**
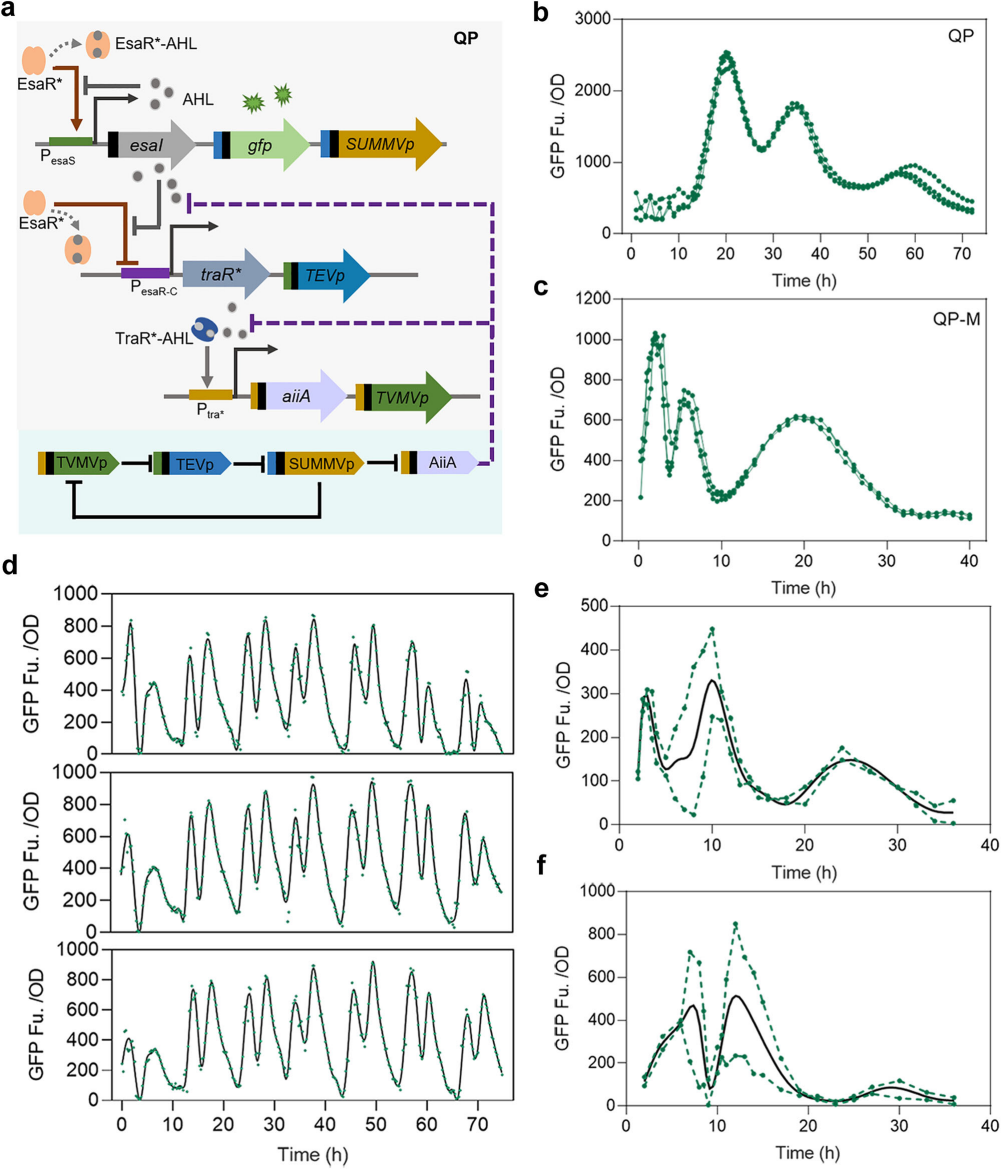
Design and characterization of QP. **a** QP circuit regulated by Esa QS-switch, cascade components and protease regulatory module. Initially, EsaR* activates the expression of EsaI, GFP, and SUMMVp controlled by P_esaS_. When AHL signals accumulate to a certain extent, the expression of EsaI and GFP would be shut down, AHL is not produced, and TraR* and TEVp controlled by P_esaR-C_ begin to express. Then, sufficient TraR* binds to AHL to activate the expression of AiiA and TVMVp controlled by P_tra*_, and AiiA degrades AHL to accelerate signal reset. Proteases provide post-translational regulation, and continuous inhibition between proteases enables rapid turnover of target proteins. **b** Fluorescence characterization of QP in 24-well plates with 1 mL of medium. The green lines are the measuring results of three independent samples. **c** Fluorescence characterization of QP-M in 24-well plates with 1 mL of medium. The green lines are the measuring results of three independent samples. **d** Fluorescence characterization of QP-M in the process of six successive periodic dilutions. The green dots are the measuring results of three independent samples. The black solid line is the fitting curve generated by GraphPad Prism software. **e** Fluorescence characterization of QP-M in shake flasks with 50 mL of medium. The green dotted lines are the measuring results of the two independent samples. The black solid line is the fitting curve generated by GraphPad Prism software. **f** Fluorescence characterization of QP-M in quadruple tanks with 400 mL of medium. The green dotted lines are the measuring results of the two groups of independent samples. The black solid line is the fitting curve generated by GraphPad Prism software.

Resetting the sensing signal is the key to achieve continuous oscillations for synthetic oscillators coupling by QS, especially in non-microfluidic environments. In our circuit, AiiA is an important factor affecting signal reset. To complete continuous oscillation, sufficient AiiA is required to degrade the AHL to reset signal to initiate the second oscillation. At the same time, AiiA should have a timely and rapid turnover to ensure that subsequent oscillations are not affected. Three programmable protein switches are designed to control protein stability and residence time by post-translational regulation^19,22^. By adjusting the direction of the proteolytic tag and the protease cleavage tag, the target protein exists stably in the absence of protease, whereas in the presence of protease, the corresponding cleavage tag is excised, exposing the proteolytic tag and leading to the rapid degradation of the target protein. We integrated the proteases regulatory module into Q3 to achieve the stable and fast turnover of AiiA (Fig. 2a). Specifically, the SUMMVp and its corresponding tag complete the post-translational regulation of AiiA, the TEVp regulates the timely emergence and degradation of SUMMVp, and the TVMVp implements the regulation of TEVp. The improved oscillation circuit was named QP. Although our initial aim was to regulate the stability and rapid degradation of AiiA, analysis of the improved system showed that not only did the regulatory levels of AiiA increase, but also the structure of the entire oscillation system changed, with the addition of a negative feedback circuit based on protease module to regulate AiiA from the original circuit. Testing circuit QP in 24-well plates and applying OD normalization to the overall fluorescence, three oscillations were observed within 70 h, with the period lengthening and the amplitude decreasing in turn (Fig. 2b).

In the subsequent repeated experiments, we found that the characterization results of the circuit QP were weak after continuous transfer. In addition, the lag phase for the bacteria was long (~10 h), and the circuit might produce a large metabolic burden. We replaced AiiA and its associated elements from a high-copy plasmid to a medium-copy plasmid, and named this modified system QP-M (Supplementary Fig. 3). Testing the circuit in 24-well plates, QP-M was able to complete three cycles within 40 h (Fig. 2c and Supplementary Fig. 4). Compared with QP, the period of QP-M was shortened, but the amplitude was also reduced. The oscillation occurred during the whole period of bacterial growth. As the bacteria grew, the period of the oscillation became longer and the amplitude decreased. The third oscillation occurred in the stationary phase, with the longest period. A recent study showed that the fraction of active ribosomes in *E. coli* reduced during periods of slow growth (including the stationary phase), thus affecting the rate of protein synthesis^49^. Therefore, the irregular oscillation may be caused by the decrease of bacteria activity during long-term culture. Continuous tests^26,50^ were performed in a microplate reader during the process of six successive periodic dilutions, and the results showed that QP-M could maintain stable oscillation behavior (Fig. 2d). Next, we attempted to test the circuit in larger volumes, such as shake flasks with 50 mL of medium and quadruple tanks with 400 mL of medium, and the circuit completed three oscillations (Fig. 2e, f). These results indicated that QP-M could perform stable oscillation behavior in non-microfluidic environments.

### The application version of the QS-protease oscillator

**Fig. 3:**
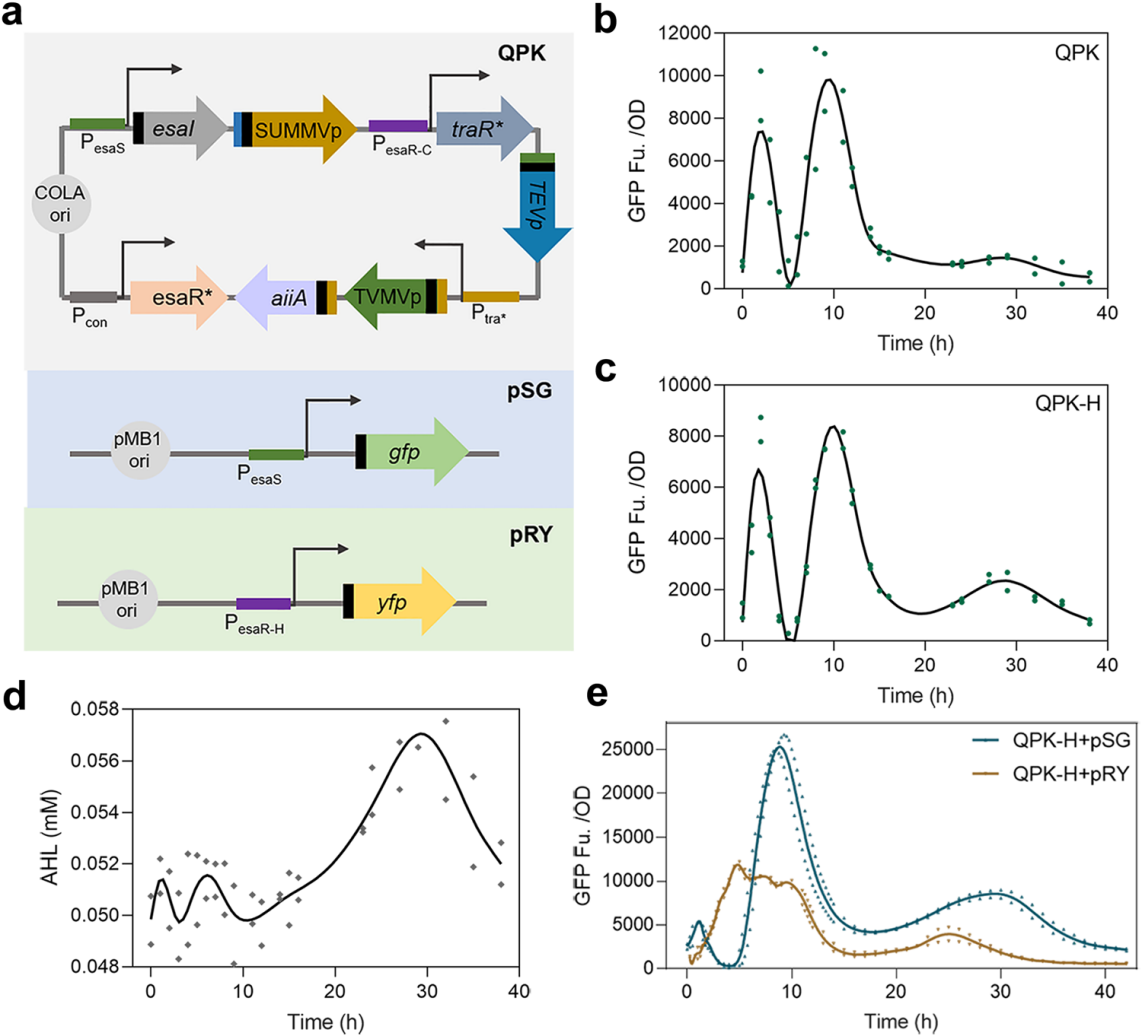
Design and characterization of QPK and QPK-H. **a** The gene composition of QPK, pSG, and pHY. **b** The result of fluorescence characterization after OD standardization of circuit QPK in shake flasks. **c** The result of fluorescence characterization after OD standardization of circuit QPK-H in shake flasks. The green dots of **b**, **c** are the measuring results of two independent samples. The solid line is the fitting curve generated by GraphPad Prism software. **d** The concentration of AHL in the process of regulation of QPK-H. **e** Characterization of reporter genes regulated by P_esaS_ and P_esaR-H_ in QPK-H circuit. The data points of **d**, **e** are the measuring results of two independent samples. The solid line is the fitting curve generated by GraphPad Prism software.

The above findings proved that circuit QP-M could realize oscillation behavior in non-microfluidic environments. We next wanted to explore the potential applications of the synthetic oscillator, but in these cases, the expression of exogenous genes on plasmids is often required. To reduce the metabolic burden from plasmids and facilitate further experimental procedures, we tried to simplify the plasmid distribution of the oscillator by assembling all regulatory elements into one plasmid, which was named QPK (Fig. 3a). In a recent study, Christina et al. constructed a hybrid promoter P_esaR-H_ with good AHL sensitivity^51^. We tried to further optimize the oscillation circuit by replacing the P_esaR-C_ promoter of QPK with P_esaR-H_, which was named QPK-H. The reporter *gfp* controlled by P_esaS_ was constructed on another plasmid, named pSG. QPK and QPK-H were then tested in shake flasks. Both were able to achieve oscillation behavior, but the third oscillation was more obvious for QPK-H (Fig. 3b, c). The concentration of signal molecules in the culture system of QPK-H was tested, and it was confirmed that the concentration of AHL fluctuated (Fig. 3d). Furthermore, P_esaR-H_, like P_esaS_, can directly respond to the changes of AHL in the regulation process of Esa QS. Thus, when P_esaS_-regulated *gfp* oscillates under the regulation of the QPK-H circuit, P_esaR-H_-regulated genes will also oscillate. To illustrate this profile, *gfp* regulated by P_esaS_ on the pSG plasmid were replaced by *yfp* regulated by P_esaR-H_, and this was named pHY (Fig. 3a). The fluorescence of pSG and pHY regulated by QPK-H was tested under the same conditions, respectively. The result revealed that the reporter genes regulated by P_esaS_ and P_esaR-H_ could complete crossing oscillations (Fig. 3e).

**Fig. 4:**
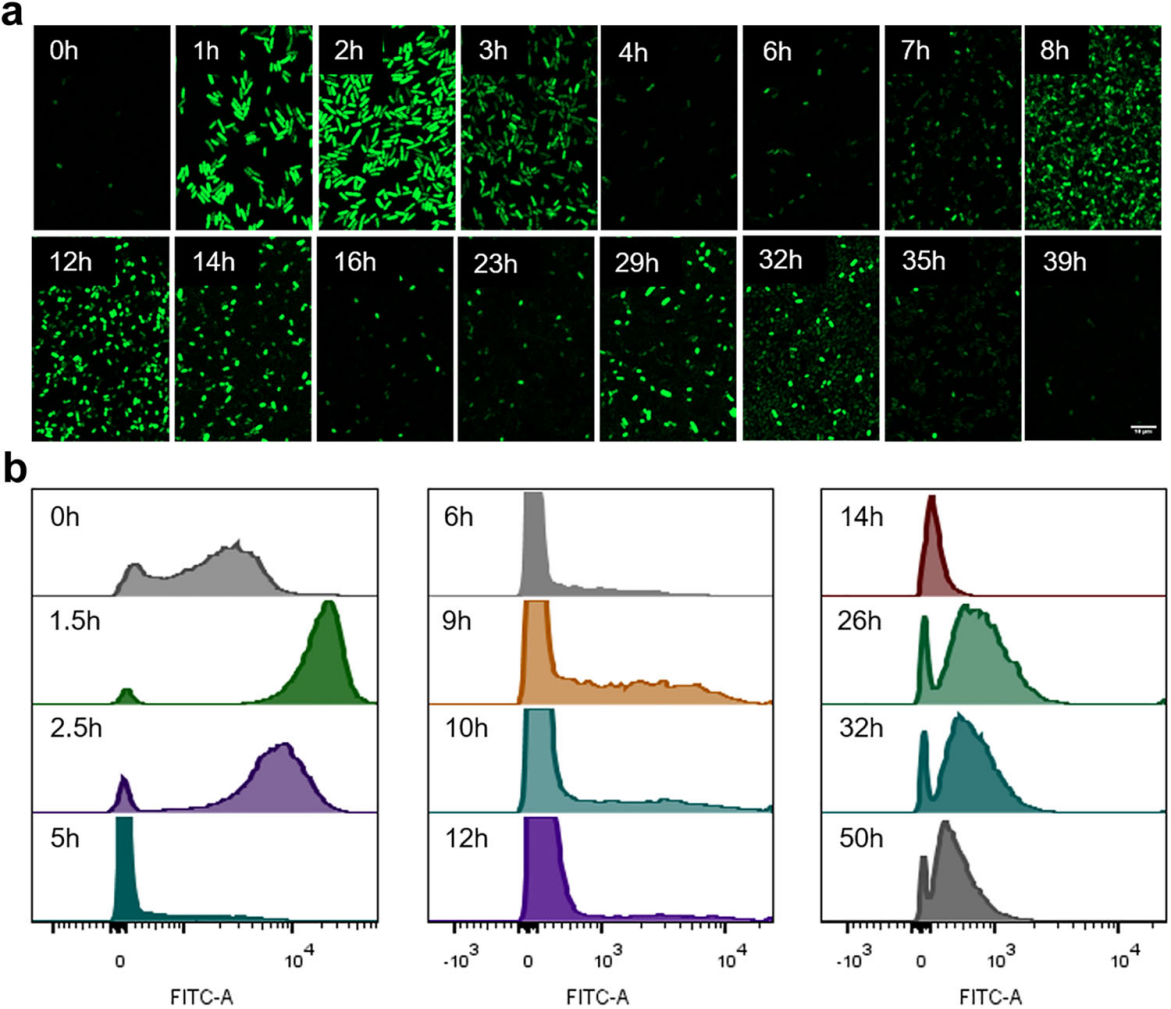
The fluorescence microscopy observation and flow cytometry analysis of QPK-H. **a** Fluorescence microscope observations of *E. coli* contained QPK-H and pSG during the process of characterization in shake flasks. The microscope magnification is 40 times. The scale bar is 10 μm. **b** Flow cytometry analysis of *E. coli* contained QPK-H and pSG during the process of characterization in shake flasks.

To further analyze the oscillations of the bacterial population, we performed fluorescence microscopy observation and flow cytometry analysis. It can be observed that the fluorescence intensity of the bacterial population fluctuated during the culture process (Fig. 4a), which was basically consistent with the data measured during the continuous culture process (Supplementary Fig. 5). Flow cytometry analysis revealed the migration of bacteria at different fluorescence intensities. Compared with the overall fluorescence intensity, the migration of bacteria could also be divided into three stages (Fig. 4b and Supplementary Fig. 6). In early logarithmic phase, the microbiota was small but active, the amplitude of the first oscillation was large and all of the bacteria completed the migration at different fluorescence intensities. In mid of the logarithmic phase, the microbiota was active and abundant, but only some bacteria needed to be involved to reach the signal threshold. Therefore, the overall fluorescence intensity of the second oscillation was obvious, but only some of the bacteria migrated. Analysis of the microbiota at fluorescence intensities from 500 to 10^5^ showed that during the second oscillation, only a subset of cells underwent rapid oscillation, with most cells remaining silent in their initial state (Supplementary Fig. 7). In stationary phase, the microbiota was large but not active, and it took a long time for the bacteria to complete the reset of signal. Therefore, the amplitude of the third oscillation was small, but most of bacteria migrated at different fluorescence intensities. Overall, QPK-H was able to accomplish population-level oscillations in shake flasks, which could be used as a regulatory circuit for various applications. There is also the potential to achieve more standardized and durable oscillations through circuit optimization in the future.

### Regulating the cell morphology of *E. coli* using the synthetic oscillator

Microbial morphology is diverse and complex, which is the result of a combination of evolutionary pressure and adaptations to natural environments and specific lifestyles. The shape and size of cells determine in part the physical properties of the cells, such as the stiffness, robustness, and surface-to-volume ratio^52,53^. The manipulation of cell morphology may therefore have an important influence over several physiological properties^54^. Synthetic morphology is valuable in understanding developmental programs and cell differentiation processes, and contribute to new and exciting practical possibilities involving the design and construction of improved microbial cell factories, such as synthetic multicellularity and chemical production^55–59^. In this section, we explored the possible role of synthetic oscillator in regulating cell morphology.

**Fig. 5:**
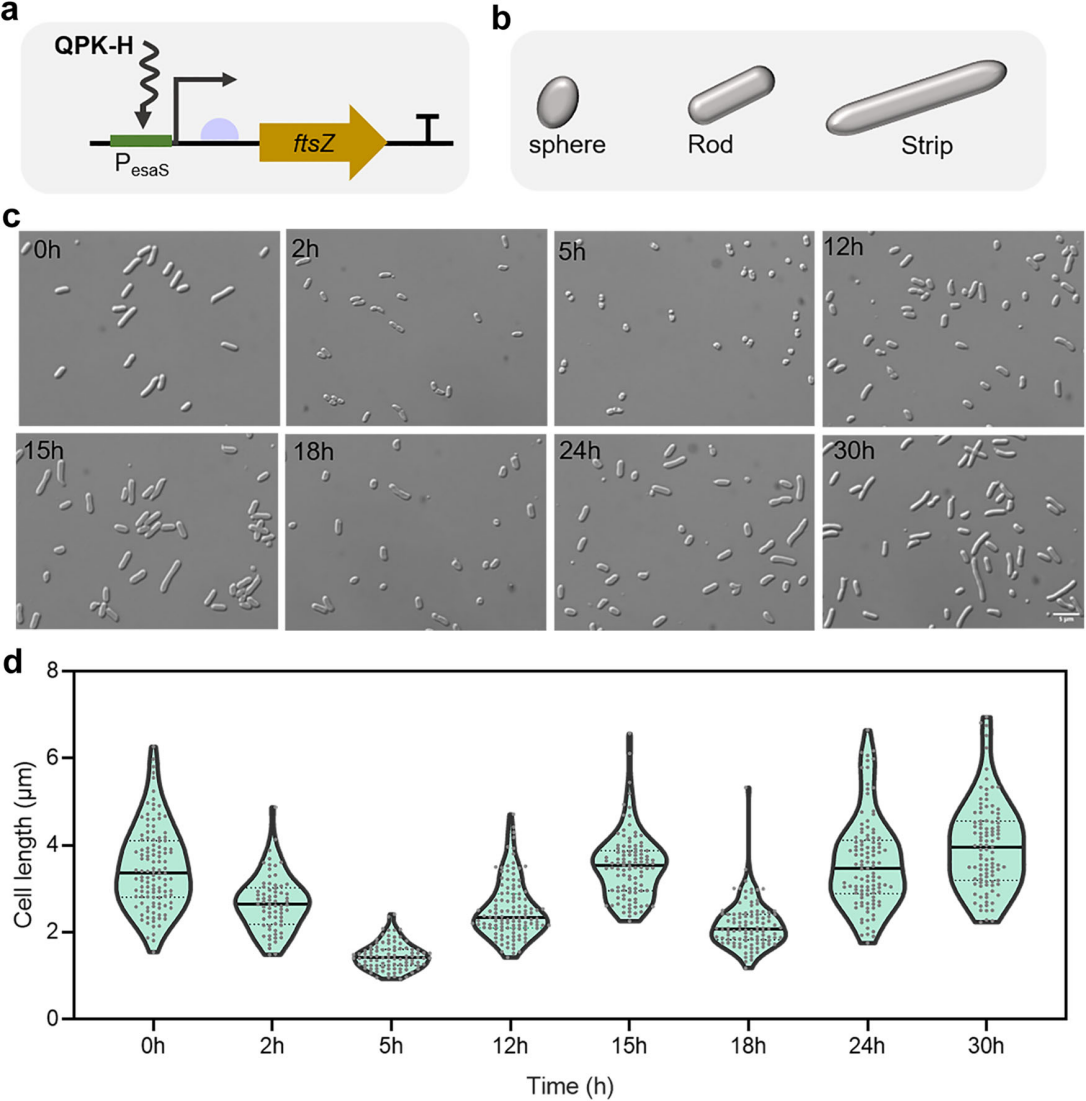
Regulating the cell morphology of *E. coli* by synthetic oscillator. **a** The periodic expression of *ftsZ*. **b** The morphology of the bacteria during the culture included sphere, rod and strip. **c** Microscopic observation of strains periodically expressing *ftsZ*. Strains were cultured in 24-well plates. The magnification is 100 times. The scale bar is 5 μm. **d** Quantitative measurements of cell lengths by ImageJ from **c**. Sample sizes of collected cells of each time point varied depending on the growth phase, 37 cells at 2 h, 60–80 cells at other time. All data points are displayed.

FtsZ, which is a tubulin-like protein, is an essential cell-division protein that is ubiquitous among bacteria^60,61^. We regulated the *ftsZ* gene via the oscillator to modulate cell morphology (Fig. 5a, b). The strains that periodically expressed *ftsZ* showed strip-rod-sphere-rod-strip changes in morphology during culture (Fig. 5c). The cell length was quantitatively measured (Fig. 5d), showing two cycles of periodic changes on the whole. In the first cycle, the changes in bacterial population were more concentrated, with the average length varying between 1.5 and 3.5 μm. While in the second cycle, the changes in the bacterial population were more dispersive, with the average length varying between 2 and 4 μm. In the control set, the average length of strain expressing only QPK-H circuit varied between 2 and 2.5 μm during culture (Supplementary Fig. 8). This case simply demonstrated that the synthetic oscillator can be used as an effective tool to regulate synthetic morphology at the population level, which would be helpful for the construction of novel cell factories or the design of living systems.

### Regulating the metabolism of *E. coli* using the synthetic oscillator

Metabolic engineering plays an important role in the construction of efficient microbial cell factories^62,63^. Regulating the periodic expression of key genes can effectively alleviate the metabolic burden of cells, but there are relatively few relevant regulatory elements. Recently, Evan et al. constructed optogenetic circuits that successfully increased iso-butanol production by regulating pathway enzyme expression by periodic light pulses^64^. The oscillation circuit constructed in this work could regulate the periodic expression of target genes at the population level, which is a potentially effective regulatory element. In this section, we demonstrated the potential application of this synthetic oscillator in regulating cellular metabolism.

**Fig. 6:**
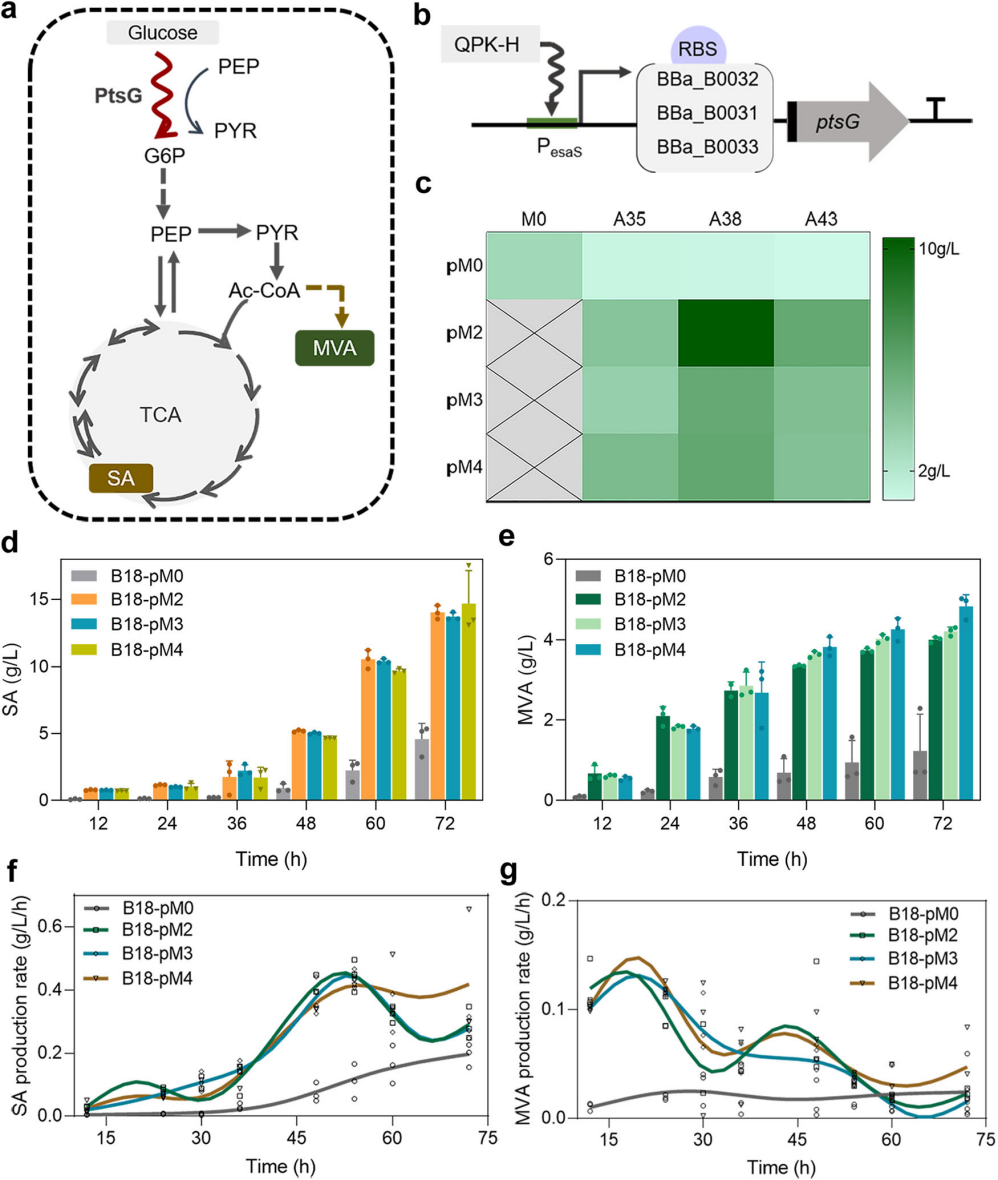
Regulating the metabolism of *E. coli* by synthetic oscillator. **a** Engineering MVA and SA biosynthetic pathway. Red curve indicated the periodic expression of *ptsG*. **b** Regulating the expression of *ptsG* with LVA degradation tag using three different strengths of RBS under the control of promoter P_esaS_. **c** The MVA yield of different engineered strains with QPK-H system at 72 h. **d** The SA yield of B18 series engineering strains at different time during fermentation. Error bars indicate three independent experiments. **e** The MVA yield of B18 series engineering strains at different time during fermentation. Error bars indicate three independent experiments. **f** The SA production rate of B18 series strains during fermentation. **g** The MVA production rate of B18 series strains during fermentation. The production rate is obtained by calculating the first-order derivative of the yield at different times. The data points are the results of three independent experiments. The fitting curve is fitted according to the data of eight time points.

In *E. coli*, the phosphotransferase system (PTS) transports glucose, which has important effects on central metabolic nodes. Modification of PTS components has a significant impact on the distribution of carbon flux in central metabolism, thereby improving or weakening the production of target products^65,66^. Studies have shown that inactivation of the *ptsG* gene reduced the glucose uptake capacity of bacteria, contributing to the accumulation of acetyl-CoA and the production of products with central metabolic intermediates as precursors^67,68^. We previously constructed an engineered strain A35, in which *ptsG* was knocked out, to produce mevalonate (MVA) generated using acetyl-CoA as the precursor (Fig. 6a). However, the MVA production was reduced by 60%, compared with the control strain MG1655 (named M0). We hypothesized that the introduction of exogenous pathway and the knockdown of endogenous gene could lead to an imbalance in metabolic flux, resulting in decreased MVA production. In this case, we attempted to regulate the periodic expression of *ptsG* by QPH-K to test the effect on MVA production. The control plasmid was pM0. Three RBS (http://parts.igem.org) with different intensities were used to regulate the expression of *ptsG* (Fig. 6b), and the production plasmids were named pM2, pM3, and pM4, respectively. The results showed that the MVA production of the three strains with *ptsG* oscillatory expression was increased but not significantly. The inactivation of pyruvate oxidase gene (*poxB*) and phosphotransacetylase gene (*pta*) is reported to promote the production of greater levels of acetyl-CoA^69^. In order to clarify the regulation effect of the oscillator on cell metabolism, we constructed strain A38 by knocking out *poxB* and strain A43 by knocking out *poxB* and *pta*, and then performed fermentation verification. The results showed that MVA production was increased in the A38 and A43 series strains with regulation of synthetic oscillator. In particular, strain A38-pM2 produced 10.4 g/L MVA, which was significantly higher than strain A38-pM0 (Fig. 6c).

We found that the A43 series strains accumulated higher levels of succinic acid (SA). The yield of SA of strain A43-pM2 was 2.5 g/L, which was 88% higher than that of the control strain A43-pM0 (Supplementary Fig. 9). Previous studies have shown that regulating glucose uptake is an important strategy to improve SA production^70^. According to previous work^68,69^, we knocked out the *sdhA* and *iclR* genes of strain A43 to construct strain B18 with the potential to produce SA. We transformed the above production plasmids into B18 strains to test the effect of oscillation circuit for the co-production of SA and MVA. The results showed that the yields of SA and MVA were increased in the experimental strain B18-pM4 to 14.70 g/L and 4.83 g/L, compared with 4.59 g/L and 1.23 g/L in the control strain B18-pM0, respectively (Fig. 6d, e). The growth and glucose consumption curves of the above strains are shown in the supplementary information (Supplementary Figs. 10 and 11).

We calculated the production rates of products during the fermentation of the B18 series engineering strains, and the production rates of SA and MVA were found to change periodically (Fig. 6f, g). This suggested that the synthetic oscillator could affect the synthesis rate of the products, which might be a feasible solution to alleviate the cell metabolic burden while improving production. In summary, this section demonstrated the positive effect of periodically regulating the expression of *ptsG* by a synthetic oscillator on improving cell growth and producing compounds related to central metabolism. Our findings provide a potential new strategy for the design of efficient cell factories.

## Discussion

In this study, we constructed a synthetic oscillator based on the regulation of QS and protease elements, achieving population-level oscillations during continuous culture in non-microfluidic environments. The oscillator was proven to be a useful tool in regulating cellular morphology and metabolism.

The motifs of synthetic oscillators have been successfully elucidated. Negative feedback with time delay can trigger oscillation, and positive feedback confers tunability to the oscillator^71^. The construction of synthetic oscillators is mostly based on these principles, such as Goodwin oscillator, Repressilator and the dual-feedback oscillator^12,13,72^. In our circuit, the accumulated QS signals complete the delayed population-level negative feedback to drive the emergence of oscillation. The mechanism is similar to the coupled Goodwin oscillator^10,12,43,44^. In synthetic oscillators, negative feedback prevents signal overload, which is necessary to carry a reaction network back to the “starting point” of its oscillation^72^. Signal reset is also crucial for coupling the oscillator to achieve continuous population-level oscillation, especially in large-scale environments. In the non-microfluidic environment, the accumulation of signal molecules will continue to increase, and the negative feedback at the transcriptional level may not be enough to complete signal reset. Therefore, we regulated the cascaded expression of AiiA to directly degrade signals to further prevent signal overload and accelerate signal reset. The activation element is designed as a measure to enhance the effect of negative feedback, similar to the effect of positive feedback in some oscillation circuits^45^. Furthermore, the components of proteases and corresponding tags are helpful in optimizing the oscillation circuit^22^. Post-translational regulation of AiiA confers more precise regulation of the circuit. The combination of transcriptional regulation and post-translational regulation is the key to achieve the continuous oscillation of the circuit in a non-microfluidic environment. At present, we have not fully sure about the system characteristics of components of the circuit, and thus it is not yet possible to simplify the circuit generically to a clear mathematical model to accurately analyze the operating principles and explore methods to modify the oscillation parameters.

The comparison of flow cytometry analysis and overall fluorescence intensity showed that the population-level oscillation in this work was not a direct rapid change, but rather a slow migration. This circuit could indeed lead to the oscillation of the overall fluorescence and affect the distribution of the bacteria at different fluorescence intensities. However, the oscillations at different stages of bacteria growth were not regular, and we speculated that this phenomenon was closely related to the growth activity and size of the bacteria population^49^. In the repeated experiments of QP-M, the two oscillations that occurred in log phase were stable, but the subsequent oscillations were more random, sometimes resulting in four oscillations and sometimes displaying decreased amplitude (Supplementary Fig. 4). The activated strains (such as retransforming plasmids into strains) could exhibit better oscillation behavior, indicating that the growth activity had some influence on the characterization of the circuit. Additional validation experiment also showed that growth status would affect the strength of gene expression (Supplementary Fig. 12). One of the characteristics of the biological clock is that it is independent of the growth rate. The biological clock ensures that the organism could complete its normal physiological process, and normal metabolism in turn ensures that cells are maintained at a relatively stable number and state to allow for regular operation of the biological clock. Coupling the synthetic clock to cell growth or exploring mechanisms to mitigate the effects of growth is important for building a robust synthetic clock.

We explored the potential applications of the synthetic oscillator in regulating cellular morphology and metabolism at the population level. More effort is still needed to optimize synthetic oscillators to match the natural clock as closely as possible. On the one hand, we should optimize the circuit structure or explore new components to enhance the stability and adjustability of the circuit. On the other hand, creating large libraries for high-throughput screening to obtain ideal circuits is an effective strategy^73,74^. Recently, researchers have addressed the technical difficulties in screening mutant libraries of population-level genetic circuits for dynamic phenotypes. A workflow is developed for quantitatively screening libraries of genetic circuits to tune the dynamics of the synchronized lysis circuit, with the final oscillator exhibiting robust and tunable oscillations over long time scales^37^.

The synthetic oscillator in non-microfluidic environments provides a simple simulation of the biological clock. In this circuit, negative feedback based on transcriptional and post-translational regulation, and coupling based on QS jointly achieve population-level oscillations. This demonstrates that robust oscillators contain both core loops and multiple levels of regulation. This is similar to the coupling principle of the natural biological clock. The biological clock of cyanobacteria and eukaryotes includes regulation at the transcriptional and post-translational levels^29,75,76^. Our work presents a synthetic oscillator that operates autonomously and continuously at the population level in non-microfluidic environments and provides useful information for the future design of large-scale, stable, synthetic clocks.

## Methods

### Strains and plasmids

Strains and key genes are listed in Supplementary Table 1. Plasmids are listed in Supplementary Fig. 13 and Supplementary Table 2. Promoters are listed in Supplementary Table 3. All constructs used for tools development were generated using ligation cloning procedures from ABclonal Technology (Wuhan, China). All knockout or integration experiments were performed using homologous recombination methods^77^.

*E. coli* MG1655 was used to characterize synthetic oscillator. The plasmids of Q2-L were pQA2 and pQEL. The plasmids of Q2-A were pQA2 and pQEA. The plasmids of Q2-T were pQA2T and pQET. The plasmids of Q3-L were pQA3 and pQEL. The plasmids of Q3-A were pQA3 and pQEA. The plasmids of Q3-T were pQA3T and pQET. The plasmids of QP were pQP1 and pQP2. The plasmids of QP-M were pQP1 and pQP2M. The plasmids of QPK were pQPK and pSG. The plasmids of QPK-H were pQPH and pSG. The promoter P_esaR_ used in this study is the P_esaR-C_ that we characterized before^42^. The promoter P_tra*_ used in this study is the variant P_tra_ that we used before^48^.

In the part of cell morphology observation experiments, the promoter of *ftsZ* on MG1655 genome was replaced by P_esaS_, and esaR* controlled by J23101 was integrated into the genome to construct the engineered strain MS101. The regulatory plasmid was pQPH-fts without esaR*. The control set was the strain expressing only oscillation circuit.

In the part of regulating metabolism experiments, the engineered strains included A35, A38, A43, and B18. In strain A35, the *ptsG* was knockout. In strain A38, the *ptsG* and *poxB* were knockout. In strain A43, the *ptsG*, *pta* and *poxB* were knockout. In strain B18, the *ptsG*, *pta*, *poxB*, *sdhA* and *iclR* were knockout to accumulate succinate. The control plasmid was pM0 to produce MVA. The experimental plasmids were pM2, pM3, pM4, with different intensity of RBS to control the expression of *ptsG* and produce MVA, respectively. The regulatory plasmid was pQPH.

### Culture conditions

Luria-Bertani (LB) broth (5 g/L yeast extract, 10 g/L tryptone, 10 g/L NaCl) was used for circuit characterization and morphologic observation, and LB agar (LB broth supplemented with 15 g/L agar powder) was used for plasmid construction and screening. To maintain plasmids, antibiotics, namely chloramphenicol (25 μg/mL), kanamycin (50 μg/mL), ampicillin (100 μg/mL), and spectinomycin (50 μg/mL), were used.

For the production of MVA and SA in shaker flasks, seed cultures were grown overnight in LB medium at 37 °C and then transferred into 50 mL fermentation medium^78^. The fermentation medium contained (g/L, unless stated) the following: Na_2_HPO_4_·12H_2_O, 17.1; KH_2_PO_4_, 3.0; NaCl, 3.0; NH_4_Cl, 1.0; Yeast extract, 5.0; Citric acid, 0.2; MgSO_4_, 1.0 mM; CaCl_2_, 0.1 mM; Thiamine hydrochloride, 0.008; D-(+)-biotin, 0.008; Nicotinic acid, 0.008; Pyridoxine, 0.032; and 1 mL/L of Trace metal solution. The Trace metal solution contained (g/L): NaCl, 10; Citric acid, 40; ZnSO_4_·7H_2_O, 1.0; MnSO_4_·H_2_O, 30; CuSO_4_·5H_2_O, 0.1; H_3_BO_3_, 0.1; Na_2_MoO_4_·2H_2_O, 0.1; FeSO_4_·7H_2_O, 1.0; and CoCl_2_·6H_2_O, 1.0. All cultures were grown at 200 rpm, 30 °C.

### Fluorescence intensity characterization

For the characterization of circuits in 24-well plates, 1% inoculum or directly inoculated in 24-well plates containing 1 mL medium. The fluorescence and cell density were measured periodically in the process of continuous culture at 30 °C by a multi-detection microplate reader (Synergy HT, Biotek, USA). For the test of circuit stability, continuously tests in a microplate reader by transferring 1% inoculum into a new 24-well plate every ~12 h.

For the characterization of circuit in shake flasks, the 1% inoculum was transferred to shake flasks containing 50 mL medium and sampled at intervals, cultured at 30 °C, 220 rpm. The fluorescence and cell density were measured by a multi-detection microplate reader. The test did not require the addition of inducers and periodic dilution.

For the characterization of circuit in quadruple tanks, the 1% inoculum was transferred to quadruple tanks containing 400 mL medium and sampled at intervals, cultured at 30 °C, 400 rpm. The fluorescence and cell density were measured by a multi-detection microplate reader.

### Cytometry analysis

Strains were cultured in shake flasks containing 50 mL of LB medium at 200 rpm, 30 °C. Samples were taken at intervals and washed and diluted to OD less than 0.1 with phosphate buffered saline. The cytometry analysis was carried out by a flow cytometer (BD, USA). Fluorescence positive cells were captured under the excitation spectrum of 488 nm (FITC channel, GFP), the channels of forward scatter (FSC) and side scatter (SSC). Furthermore, cells were first gated by FSC and SSC to illuminate noise events. Subsequently, fluorescence positive events were determined by fluorescence channels of FITC. Finally, cytometer data were processed and analyzed by FlowJo software for generating the mean value of fluorescence intensity. The negative control group was *E. coli* MG1655. The gating strategy is shown in Supplementary Fig. 14.

### Fluorescence microscope observation

Strains were cultured in shake flasks containing 50 mL of LB medium at 200 rpm, 30 °C. Samples were taken at intervals. Concentrate OD of the sample to about 0.8 (slight turbidity is enough) with phosphate buffered saline. Agarose gel (20 mL ddH_2_O, 0.1 g agarose, 0.5 g LB powder) was prepared, melted and poured into a plate. The thickness of the gel was about 1 mm and cut into a square of about 1.5 cm. 5 μL of bacteria solution were dropped onto a long cover glass and the gel was applied to the sample for inverted microscope observation. Microscopy images of samples were taken using a Super Resolution Laser Scanning Confocal Microscope (LSM900). The magnification was 40 times. GFP fluorescence intensity was analyzed using ImageJ software.

### Microscopic observation of cell morphology

Strains were cultured in 24-well plates containing 1 mL of LB medium. 3 μL samples were taken at intervals. The cell shape was recorded by bright-field imaging using Positive fluorescence microscope at 100 magnification times. Cell lengths were measured by ImageJ manually.

### Analysis of chemical concentrations

OD was measured at 600 nm with a spectrophotometer (Shimadzu, Japan). Fermentation samples were centrifuged at 12,000 rpm for 5 min and the supernatant was used for extracellular metabolite detection. Glucose, succinate, and MVA were quantitatively determined using an HPLC system (Shimadzu, Japan) equipped with a refractive index detector (RID-10A; Shimadzu) and an Aminex HPX-87H ion exclusion column (Bio-Rad, USA). AHL was quantitatively determined using an HPLC system equipped with a variable wavelength detector and a SHIMADZU FU-ODS chromatographic column.

### Statistics and reproducibility

The details about experimental design and statistics used in different data analyses performed in this study are given in the respective sections of results and methods. In the characterization of circuit, the results of two or three independent samples are usually presented simultaneously, and the results of characterization and fitting have been shown in the manuscript. In fermentation experiment, error bars indicate three independent experiments. The analyses are performed using the GraphPad Prism software.

### Reporting summary

Further information on research design is available in the Nature Portfolio Reporting Summary linked to this article.

## Supplementary information

Liang_Peer Review File

Supplementary Information

Description of Additional Supplementary Files

Supplementary Data 1

Supplementary Data 2

Reporting Summary

## Data Availability

Data supporting the findings of this work are available within the paper and its Supplementary Information files. A reporting summary for this article is available as a Supplementary Information file. The source data underlying Figs. 1d–i, 2b–f, 3b–e, 4a, 5c, d, and 6c–g, as well as Supplementary Figs. 1c, d, 2, 4, 5, and 8–12 are provided in Supplementary Data 1. The key plasmids containing oscillation circuit developed in this study are provided in Supplementary Data 2. The other relevant data during the current study is available from the corresponding author upon request.
