# Supplementary material for: A synthetic population-level oscillator in non-microfluidic environments
Source: Commun Biol. 2023 May 13;6:515. doi: 10.1038/s42003-023-04904-0 (PMC10183009; doi:10.1038/s42003-023-04904-0)
Supplement: Supplementary file 2 — Supplementary Information [file 42003_2023_4904_MOESM2_ESM.pdf]

Supplementary information

A synthetic population-level oscillator in non-microfluidic environments

Fei Gu[1], Wei Jiang[2], Fangbing Kang[1], Tianyuan Su[1], Xiaoya Yang[1],

Qingsheng Qi[1]*, Quanfeng Liang[1]*

[1] State Key Laboratory of Microbial Technology, Shandong University, No. 72, Binhai Road, Qingdao 266237, China

[2] Research Center of Basic Medicine, Central Hospital Affiliated to Shandong First Medical University, Jinan, China

* Corresponding author:

Quanfeng Liang: liangquanfeng@sdu.edu.cn;

Qingsheng Qi: qiqingsheng@sdu.edu.cn

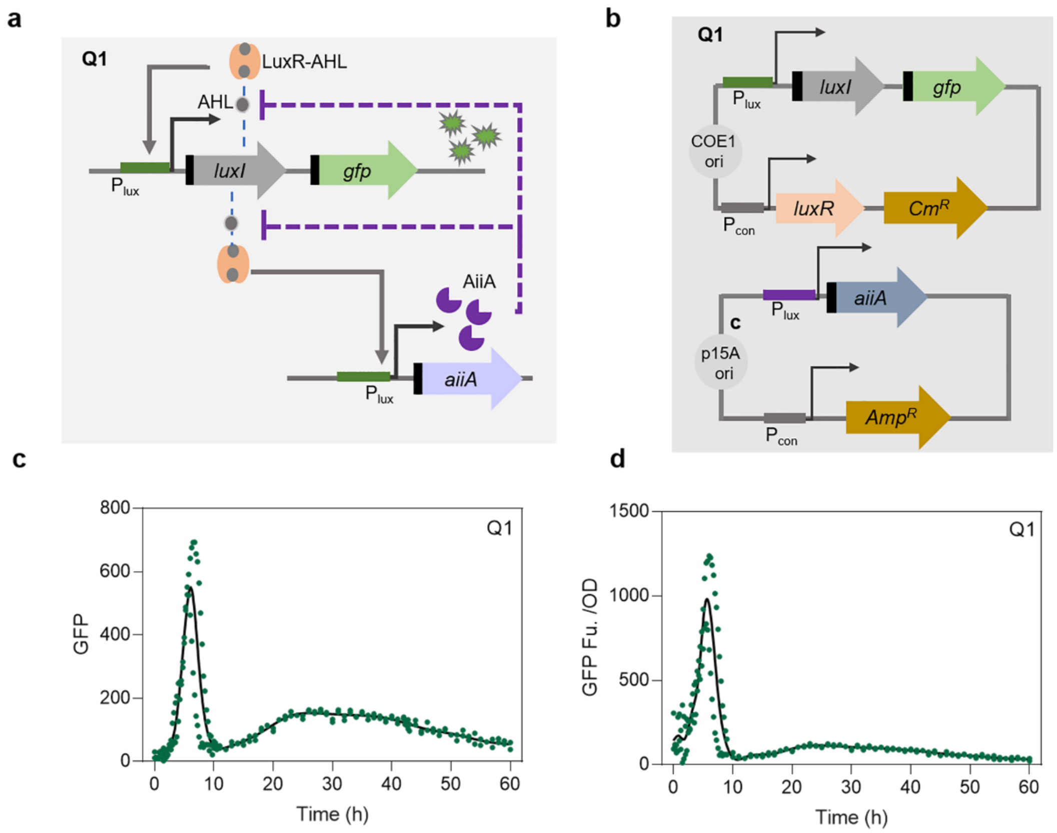

Supplementary Fig. 1. Reconstruction of the synchronized genetic clock. **a** The Schematic diagram of synchronous genetic clock, named Q1. **b** The gene distribution of this circuit. The black label indicates a degradation tag. The degradation tag of LuxI and GFP is LAA. The degradation tag of AiiA is DAS. **c** The overall fluorescence characterization of Q1 in 24 well plates. **d** The fluorescence result after OD standardization of Q1 in 24 well plates. The green dots are the measuring results of two groups of independent samples. The black solid line is the fitting curve generated by GraphPad Prism software.

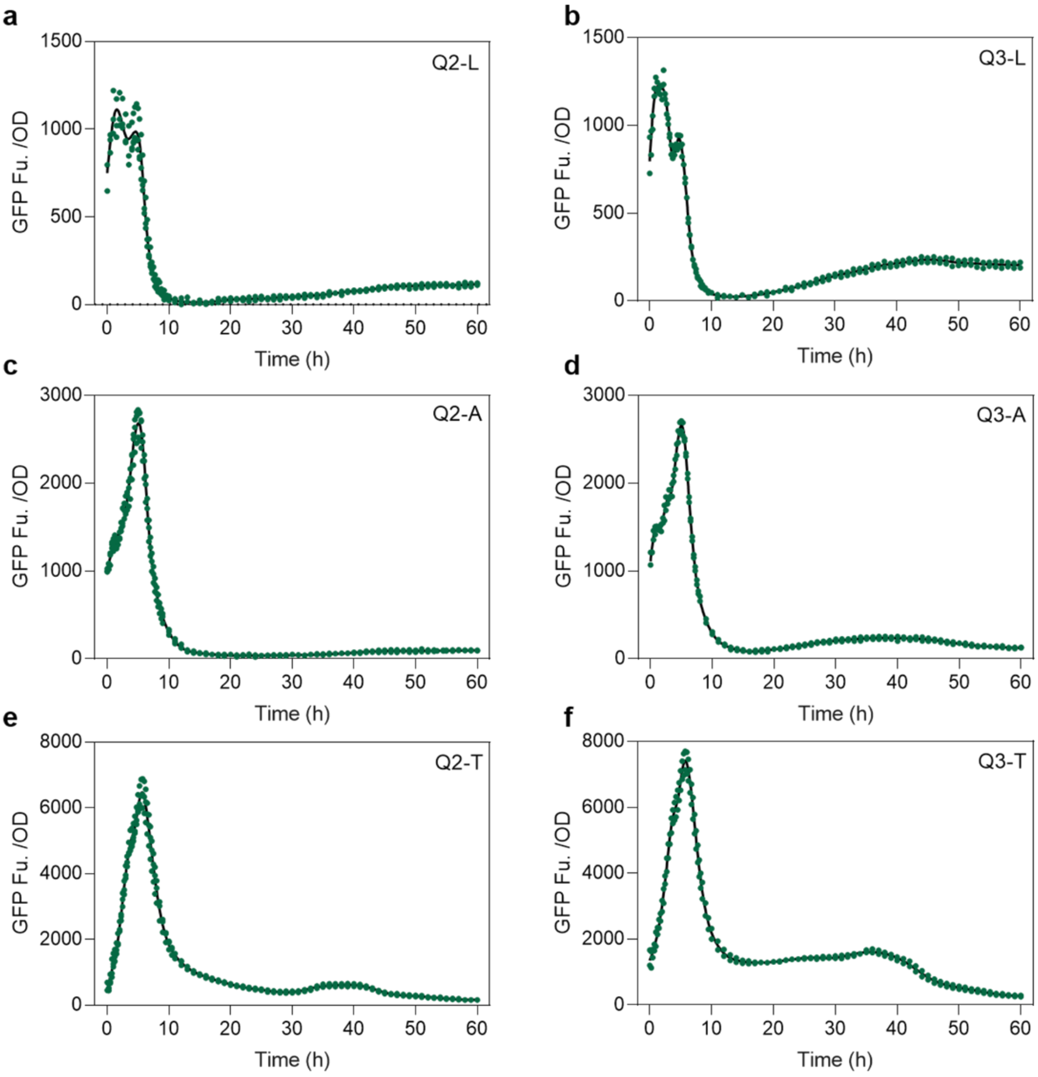

Supplementary Fig. 2. The fluorescence characterization results after OD standardization of Q2-L, Q2-A, Q2-T, Q3-L, Q3-A, Q3-T in 24 well plates. The green dots are the measuring results of two groups of independent samples. The black solid line is the fitting curve generated by GraphPad Prism software.

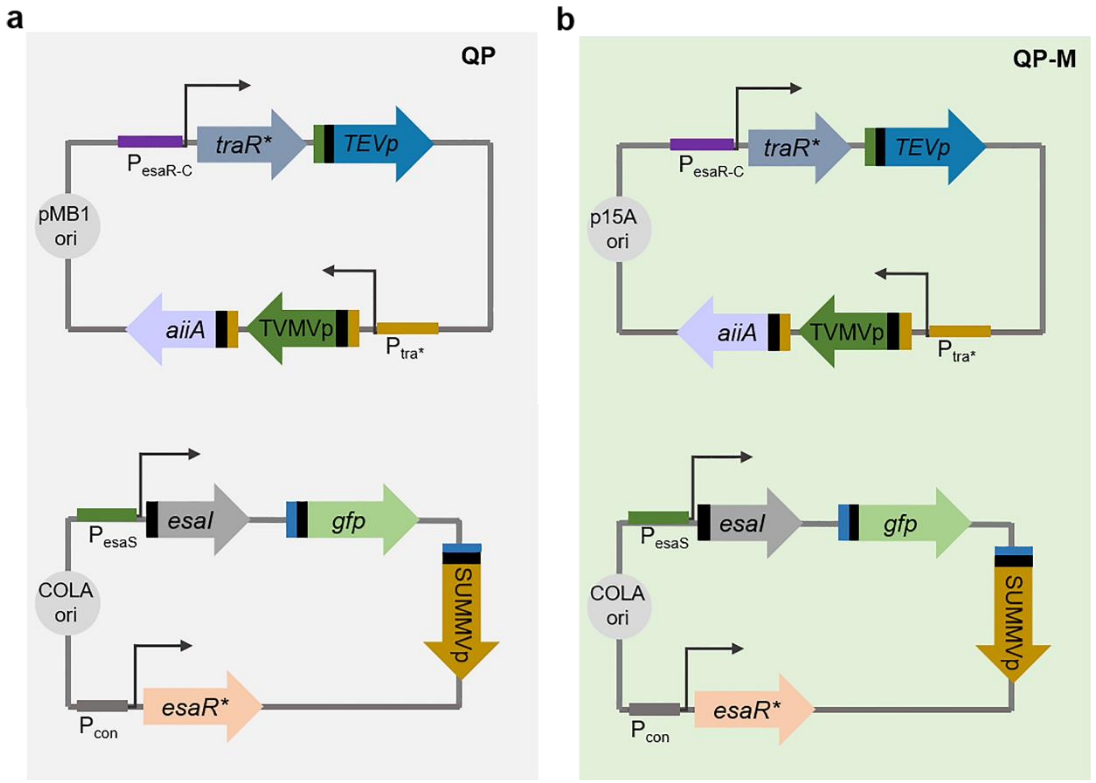

Supplementary Fig. 3. The gene distribution of circuit QP and QP-M. The genetic elements of circuit QP-M were all located in the medium-copy plasmid.

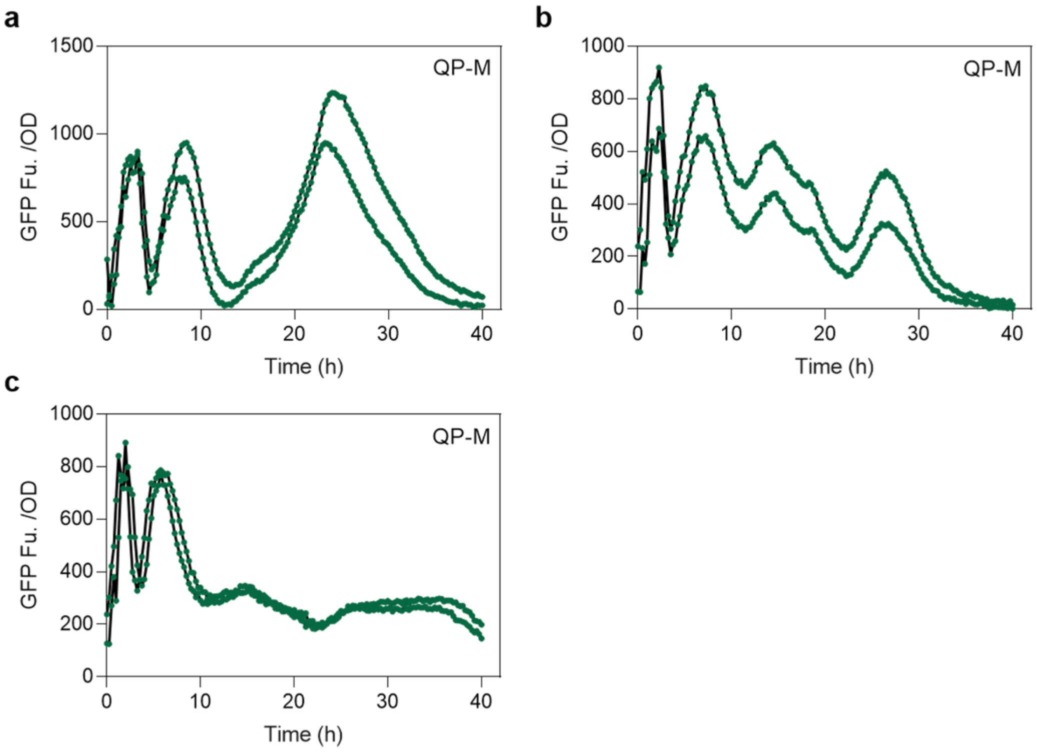

Supplementary Fig. 4. The repeated characterization of QP-M in 24-well plates. The circuit could maintain oscillation during the active growth period, that is, the first two oscillations were stable, but the subsequent oscillations might not be maintained. The lines are the measuring results of two independent samples.

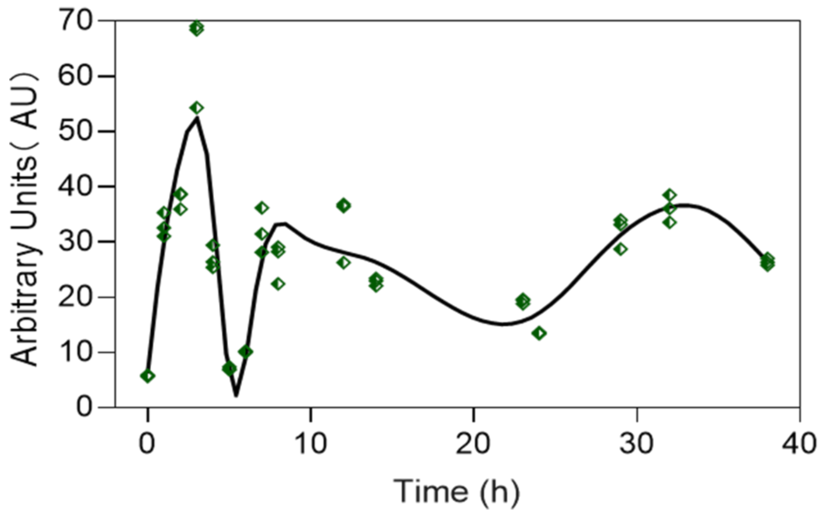

Supplementary Fig. 5. The change of fluorescence intensity in the experiment was observed by

fluorescence microscope. The fluorescence intensity of at least three pictures in each group was measured by ImageJ.

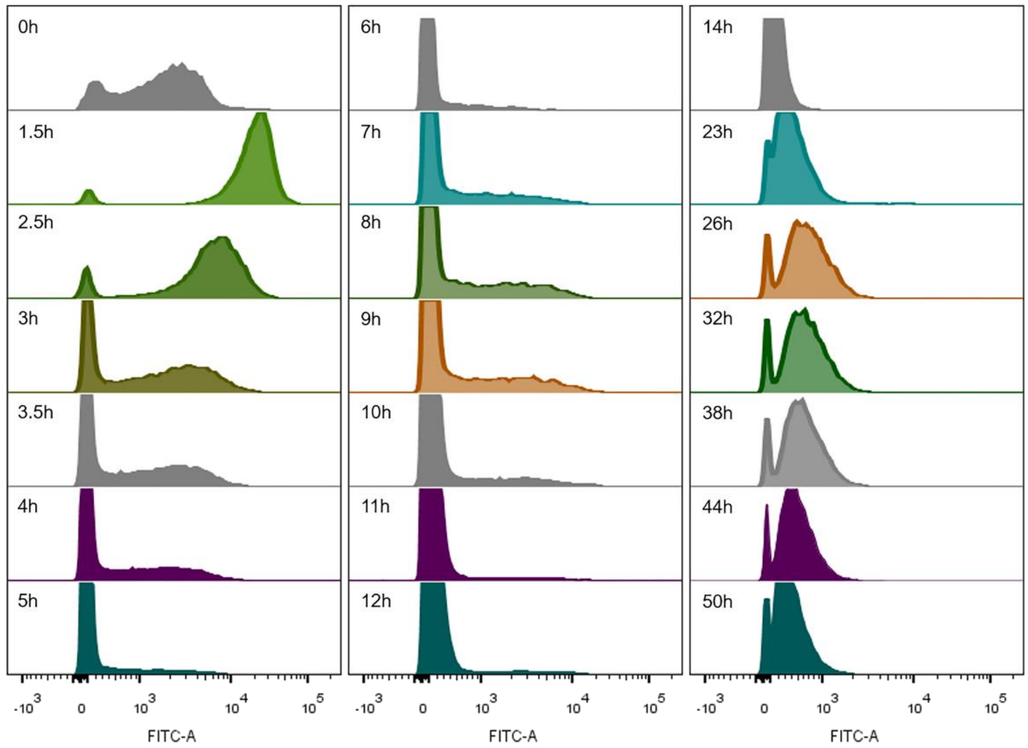

Supplementary Fig. 6. Flow cytometry analysis of QPK-H during the process of characterization in shake flasks.

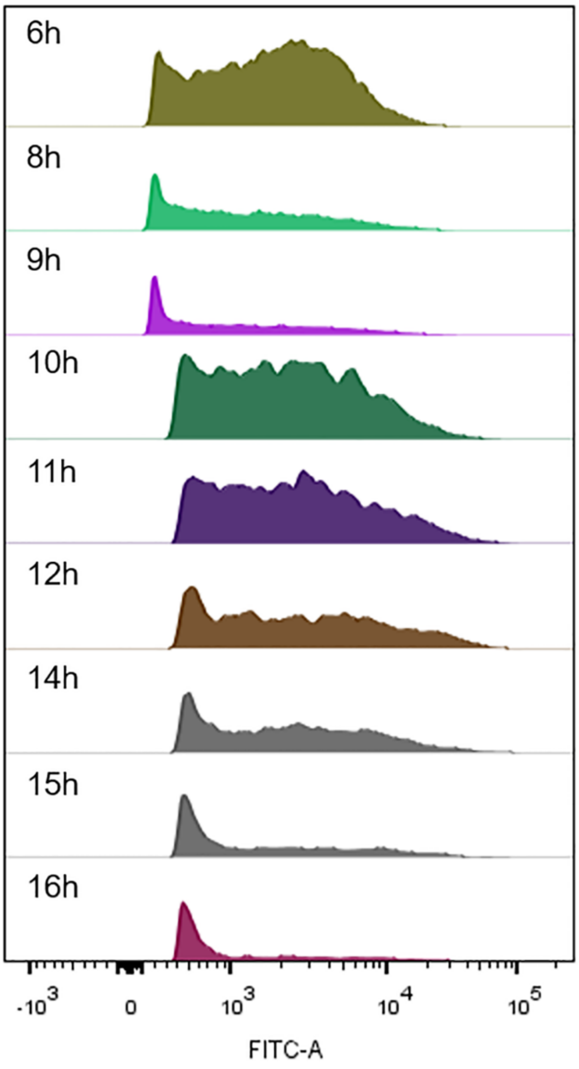

Supplementary Fig. 7. The change of fluorescence intensities from 500 to 10^5 during the second oscillation. Only some of the cells rapidly completed the oscillation behavior, and most of the cells remained silent at the initial state.

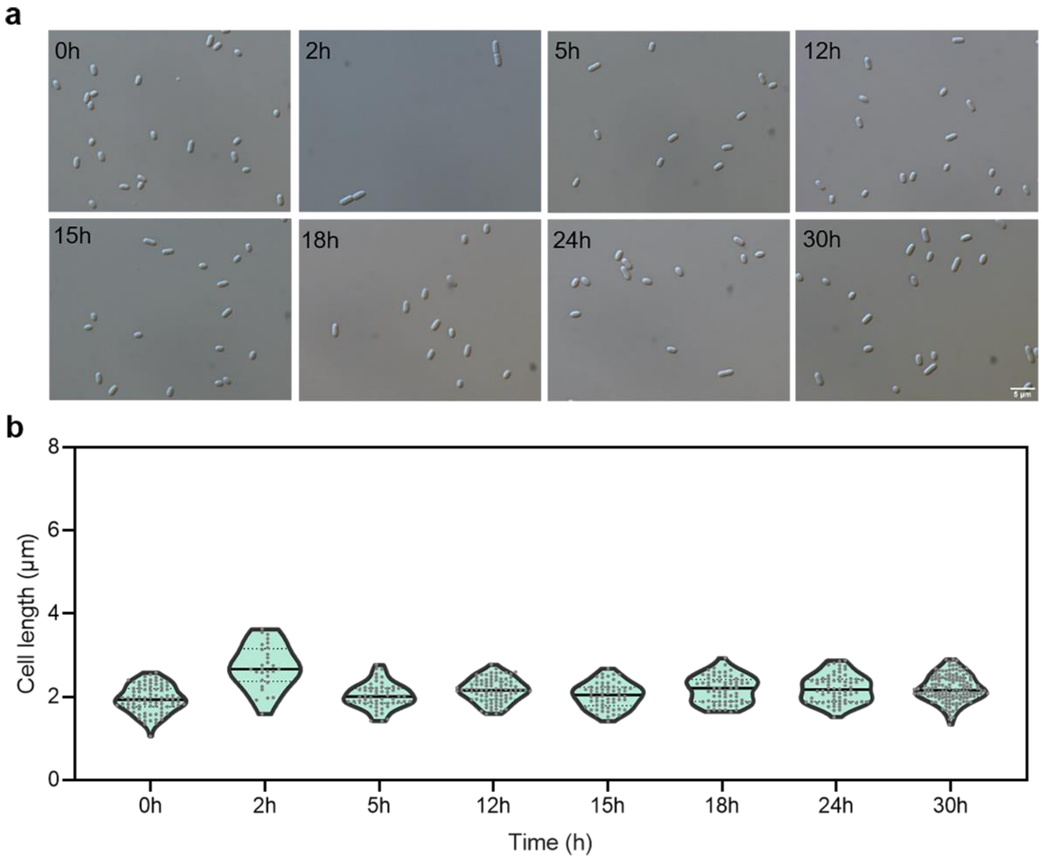

Supplementary Fig. 8. **a** Morphological observations of MG1655 expressing only the QPK-H circuit during culture. The scale bar is 5 μm. **b** Quantitative measurements of cell lengths by ImageJ from part a. Sample sizes of collected cells of each time point varied depending on the growth phase, 29 cells at 2h, 50-70 cells at 5h, 15h, 18h, and 24h, 80-140 cells at other time . All data points are displayed.

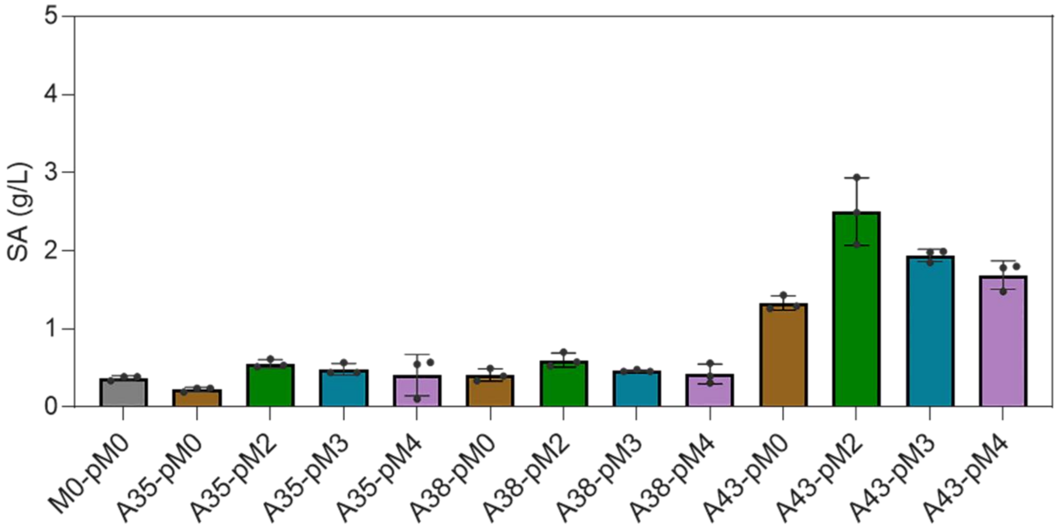

Supplementary Fig. 9. The SA yield of A35, A38, and A43 series engineering strains. Error bars indicate three independent experiments..

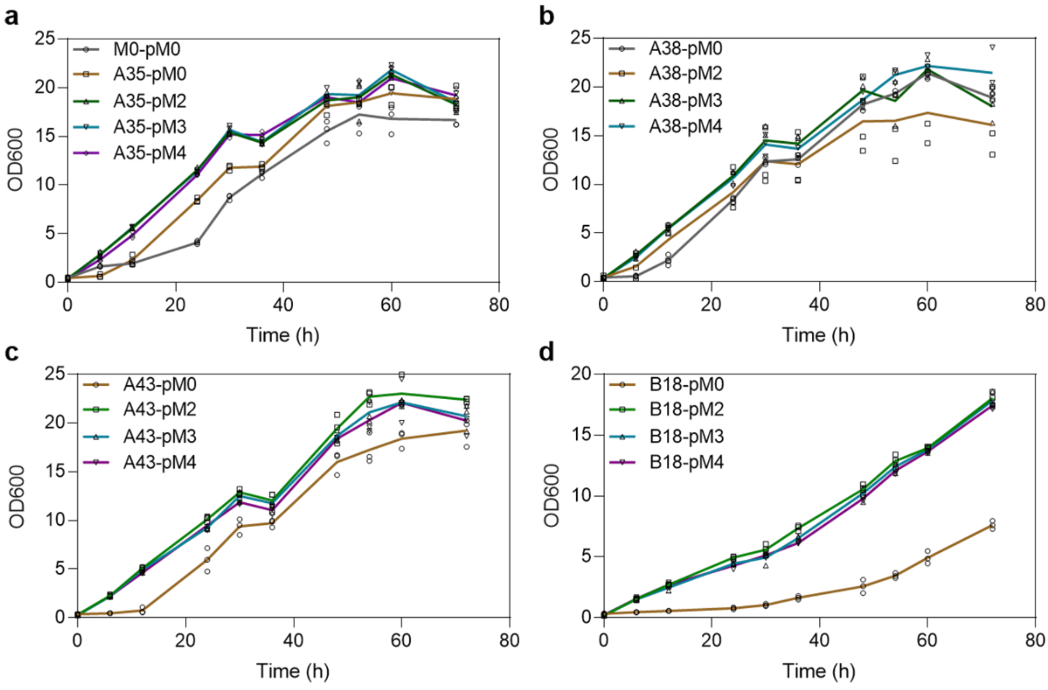

Supplementary Fig. 10. The growth curve of A35, A38, A43, B18 series engineering strains. . The data points are the results of three independent experiments.

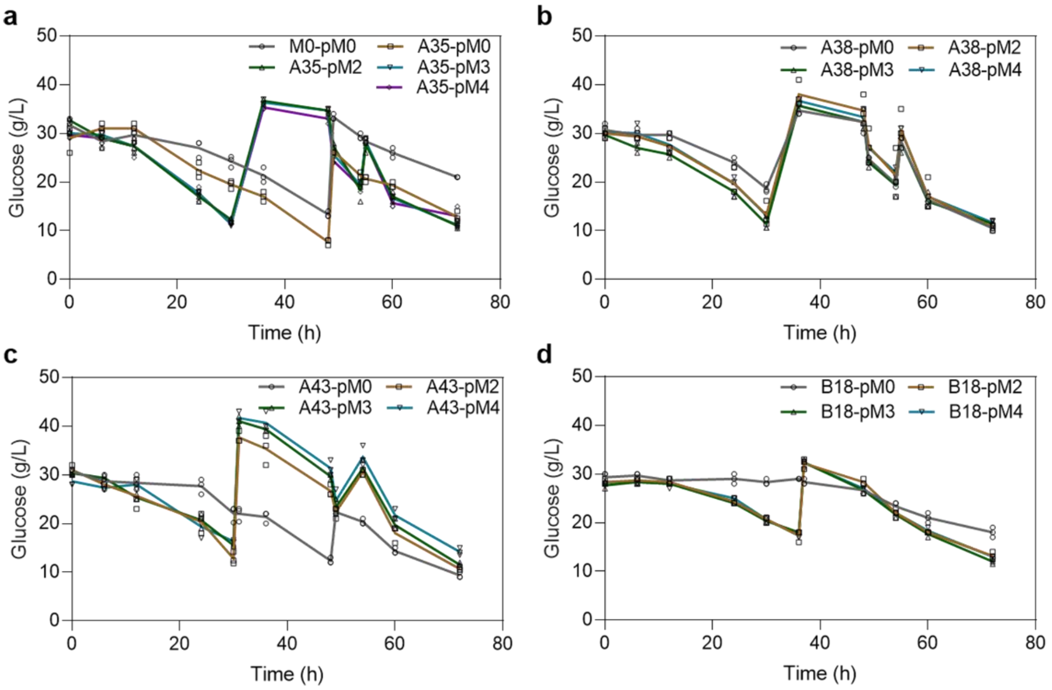

Supplementary Fig. 11. The glucose consumption of A35, A38, A43, B18 series engineering strains. The data points are the results of three independent experiments.

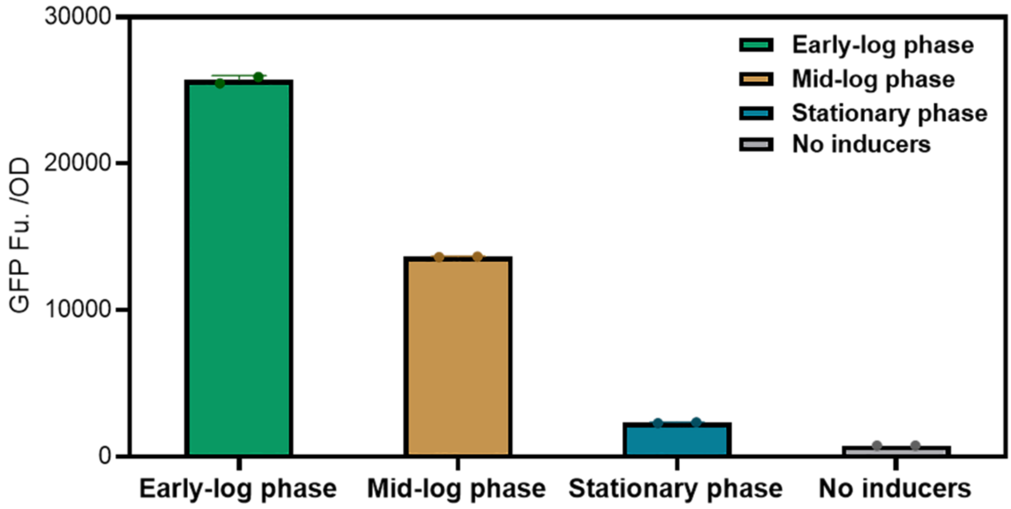

Supplementary Fig. 12. The fluorescence intensity of GFP, which was induced at the early log phase, mid log phase and stationary phase. The intensity of GFP induced at the stationary phase was reduced compared with the other two groups. Error bars indicate two independent experiments.

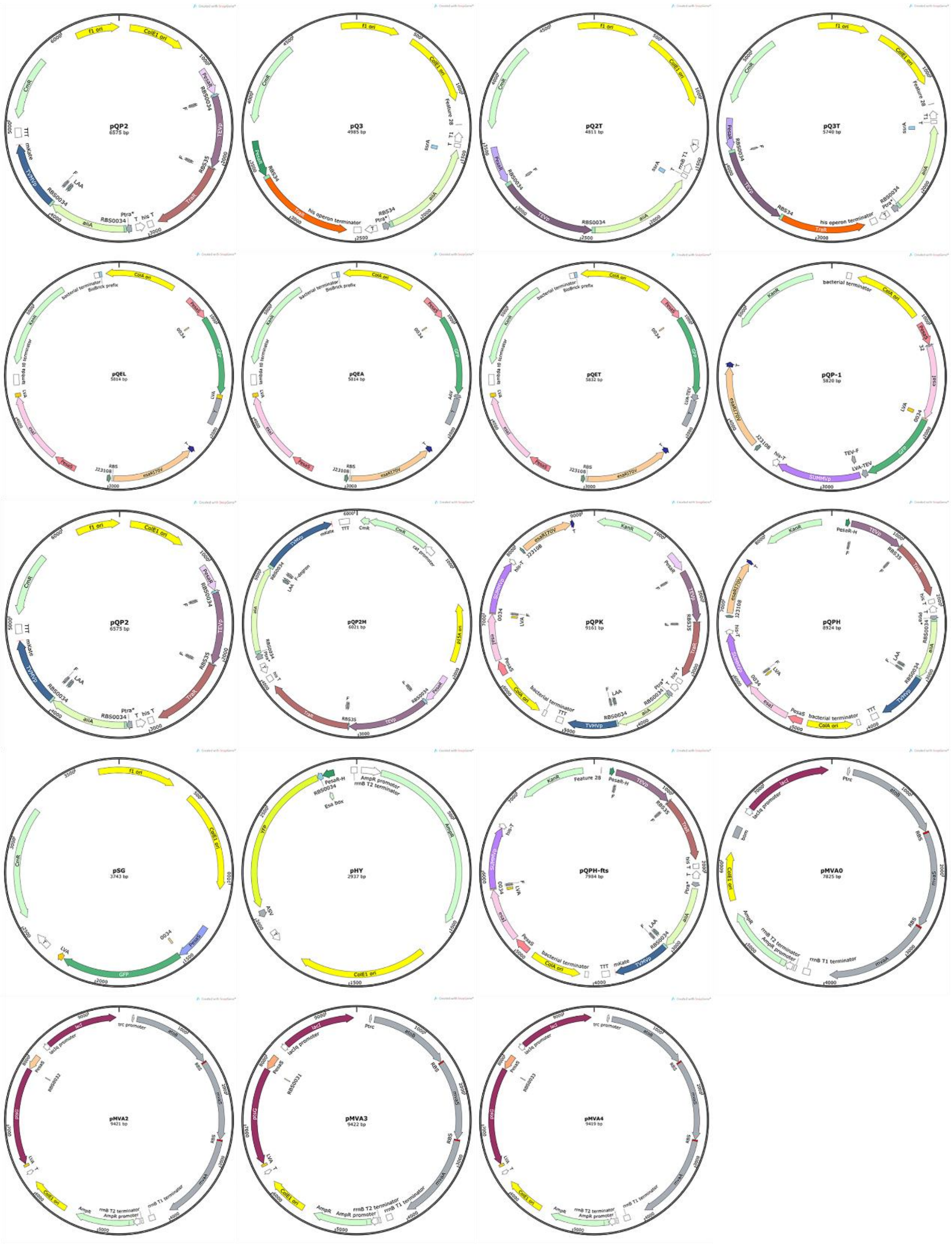

Supplementary Fig. 13. Map of key plasmids used in this study. Drawing by SnapGene software.

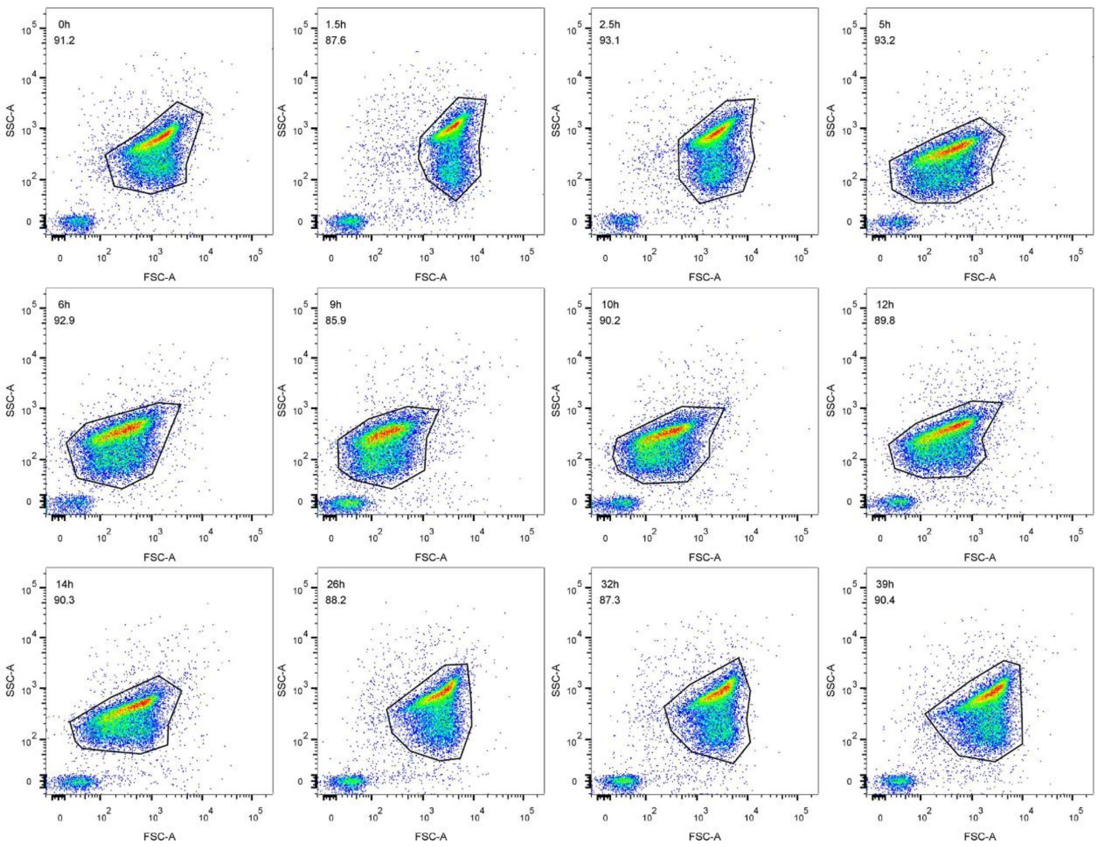

Supplementary Fig. 14. The gating strategy of flow cytometry analysis of Fig. 4b.

Supplementary Table 1 Strains and genes in this study

Stains/Genes	Description	References
strains		
E. coli MG1655	K12 F- *lambda- ilvG- rfb-50 rph-1*	Lab stock
A35	MG1655Δ*ptsG*	Lab stock
A38	MG1655Δ*ptsG*Δ*poxB*	This study
A43	MG1655Δ*ptsG*Δ*poxB*Δ*pta*	This study
B18	MG1655Δ*ptsG*Δ*poxB*Δ*pta*Δ*sdhA*Δ*iclR*	This study
MS101	MG1655 P_{ftsZ}*::Cm-* P_{esaS} *lacI::J23101-esaRI70V*	This study
genes		
esaI	Encoding a AHL synthase of Esa QS	[1]
esaRI70V	Encoding a transcription factor of Esa QS	[1]
aiiA	Encoding an AHL degrading enzyme	[2]
traR varient	Encoding a transcription factor of Tra QS	[3]
TEVp	Encoding TEV protease	[4]
TVMVp	Encoding TVMV protease	[4]
SUMMVp	Encoding SUMMV protease	[4]
atoB	The gene in the MVA production pathway	[5]
mvaS	The gene in the MVA production pathway	[5]
mvaA	The gene in the MVA production pathway	[5]

Supplementary Table 2 Plasmids used in this study

Plasmids	Relevant characteristic
pQ2	ColE1-Cm-P$_{\text{esaR-C}}$-aiiA(LAA)
pQ3	ColE1-Cm-P$_{\text{esaR-C}}$-traR*-P$_{\text{tra*}}$-aiiA(LAA)
pQ2T	ColE1-Cm-P$_{\text{esaR-C}}$-(F)TEVp-aiiA(LAA)
pQ3T	ColE1-Cm-P$_{\text{esaR-C}}$-(F)TEVp-traR*-P$_{\text{tra*}}$-aiiA(LAA)
pQEL	ColA1-Kan-J23108-esaRI70V-P$_{\text{esaS}}$-esaI(LVA)-P$_{\text{esaS}}$-gfp(LVA)
pQEA	ColA1-Kan-J23108-esaRI70V-P$_{\text{esaS}}$-esaI(LVA)-P$_{\text{esaS}}$-gfp(AAV)
pQET	ColA1-Kan-J23108-esaRI70V-P$_{\text{esaS}}$-esaI(LVA)-P$_{\text{esaS}}$-gfp(LVA-TEVtag)
pQP1	ColA1-Kan-J23108-esaRI70V-P$_{\text{esaS}}$-esaI(LVA)-gfp(LVA-TEVtag)-(TEVtag-F)SUMMVp
pQP2	ColE1-Cm-P$_{\text{esaR-C}}$-(TVMVtag-F) TEVp-(TVMVtag-F) traR*-P$_{\text{tra*}}$-aiiA(LAA-SUMMVtag)-(SUMMVtag-F) TVMVp
pQP2M	15A-Cm-P$_{\text{esaR-C}}$-(TVMVtag-F) TEVp-(TVMVtag-F) traR*-P$_{\text{tra*}}$-aiiA(LAA-SUMMVtag)-(SUMMVtag-F)TVMVp
pQPK	ColA-Kan-J23108-esaR*-P$_{\text{esaS}}$-esaI(LVA)-(TEVtag-F)SUMMVp-P$_{\text{esaR-C}}$-(TVMVtag-F)TEVp-(TVMVtag-F) traR*-P$_{\text{tra*}}$-aiiA(LAA-SUMMVtag)-(SUMMVtag-F) TVMVp
pQPH	ColA-Kan-J23108-esaR-P$_{\text{esaS}}$-esaI(LVA)-(TEVtag-F) SUMMVp -P$_{\text{esaR-H}}$-(TVMVtag-F) TEVp -(TVMVtag-F) traR*-P$_{\text{tra*}}$-aiiA(LAA-SUMMVtag)-(SUMMVtag-F) TVMVp
pSG	ColE1-Amp-P$_{\text{esaS}}$-GFP(LVA)
pHY	ColE1-Amp-P$_{\text{esaR-H}}$-YFP(ASV)
pQPH-fts	ColA-Kan-P$_{\text{esaS}}$-esaI(LVA)-(TEVtag-F) SUMMVp -P$_{\text{esaR-H}}$-(TVMVtag-F) TEVp -(TVMVtag-F) traR*-P$_{\text{tra*}}$-aiiA(LAA-SUMMVtag)-(SUMMVtag-F)TVMVp
pM0	ColE1-Amp-P$_{\text{trc}}$-atoB-mvaS-mvaA
pM2	ColE1-Amp-P$_{\text{trc}}$-atoB-mvaS-mvaA -P$_{\text{esaS}}$-RBS0032-ptsG(LVA)
pM3	ColE1-Amp-P$_{\text{trc}}$-atoB-mvaS-mvaA -P$_{\text{esaS}}$-RBS0031-ptsG(LVA)
pM4	ColE1-Amp-P$_{\text{trc}}$-atoB-mvaS-mvaA -P$_{\text{esaS}}$-RBS0033-ptsG(LVA)

Supplementary Table 3 Promoters used in this study

Promoters	Sequence
P_{esaS}	GCCAGGATCCGAATTCGCTCACAACAGTGTAAGCGTATCCGTTATTGTTTGATTTTCAAGGAAAAAAGAAAACATTCAGGCTCCATGCTGCTTCTTTTACTTAACGTGGACTTAACCTGCACTATAGTACAGGCAAGATGATACTTAAGAGTAACTTACAATGAATCATTCAGAGGTTACAATGGCTTCAGTTGTTTAGCCAATTCATT
$P_{esaR\text{-}C}$	ctcgaggcagattgagtaaccgtgaatgtttgtacaaatgtttcaaagatgttactatgagtgtcccggccagcatcactttatattttgtgaagtctggccggacgttttccctagtgttggctgttttagcgacctggccgtacaggtcaggtttttttttaccgctaaacaactgaagccattgtaacctctgaatgattcattgtaagcctgtactatagtgcaggttgcctgtactatagtgcaggttaagtccacgttaagtaaaagaagcagcggatcc
$P_{esaR\text{-}H}$	TTGACAATTAATCATCCGGCTCGTATAATGTGTGGGCCTGTACTATAGTGCAGGT
P_{tra*}	GCACGTGCAGATCTGCACATTTACGCAAGAAAATGGTTTGTTATAGTCGAATAT
J23108	tgacagctagctcagtcctaggtataatgctagc
J23101	tttacagctagctcagtcctaggtattatgctagc

Supplementary References

1. Gupta, A., Reizman, I.M.B., Reisch, C.R. & Prather, K.L.J. Dynamic regulation of metabolic flux in engineered bacteria using a pathway-independent quorum-sensing circuit. *Nat Biotechnol* **35**, 273-279 (2017).
2. Danino, T., Mondragon-Palomino, O., Tsimring, L. & Hasty, J. A synchronized quorum of genetic clocks. *Nature* **463**, 326-330 (2010).
3. Jiang, W. *et al.* Two Completely Orthogonal Quorum Sensing Systems with Self-Produced Autoinducers Enable Automatic Delayed Cascade Control. *Acs Synth Biol* **9**, 2588-2599 (2020).
4. Gao, C. *et al.* Programmable biomolecular switches for rewiring flux in *Escherichia coli*. *Nat Commun* **10**, 3751 (2019).
5. Zhu, Y. *et al.* Development of bifunctional biosensors for sensing and dynamic control of glycolysis flux in metabolic engineering. *Metab Eng* **68**, 142-151 (2021).
