# Supplementary material for: A synthetic population-level oscillator in non-microfluidic environments
Source: Commun Biol. 2023 May 13;6:515. doi: 10.1038/s42003-023-04904-0 (PMC10183009; doi:10.1038/s42003-023-04904-0)
Supplement: Supplementary file 3 — Description of Additional Supplementary Files [file 42003_2023_4904_MOESM3_ESM.pdf]

Description of Additional Supplementary Files

File name: Supplementary Data 1
Description: The source data for the graphs in the paper.

File name: Supplementary Data 2
Description: The sequences of key plasmids.
