# Supplementary material for: A synthetic population-level oscillator in non-microfluidic environments
Source: Commun Biol. 2023 May 13;6:515. doi: 10.1038/s42003-023-04904-0 (PMC10183009; doi:10.1038/s42003-023-04904-0)
Supplement: Supplementary file 6 — Reporting Summary [file 42003_2023_4904_MOESM6_ESM.pdf]

nature portfolio

Corresponding author(s): Qingsheng Qi and Quanfeng Liang

Last updated by author(s): Mar 31, 2023

Reporting Summary

Nature Portfolio wishes to improve the reproducibility of the work that we publish. This form provides structure for consistency and transparency in reporting. For further information on Nature Portfolio policies, see our Editorial Policies and the Editorial Policy Checklist.

Statistics

For all statistical analyses, confirm that the following items are present in the figure legend, table legend, main text, or Methods section.

n/a	Confirmed	
☐	☒	The exact sample size (*n*) for each experimental group/condition, given as a discrete number and unit of measurement
☐	☒	A statement on whether measurements were taken from distinct samples or whether the same sample was measured repeatedly
☒	☐	The statistical test(s) used AND whether they are one- or two-sided<br>*Only common tests should be described solely by name; describe more complex techniques in the Methods section.*
☒	☐	A description of all covariates tested
☒	☐	A description of any assumptions or corrections, such as tests of normality and adjustment for multiple comparisons
☒	☐	A full description of the statistical parameters including central tendency (e.g. means) or other basic estimates (e.g. regression coefficient) AND variation (e.g. standard deviation) or associated estimates of uncertainty (e.g. confidence intervals)
☒	☐	For null hypothesis testing, the test statistic (e.g. *F*, *t*, *r*) with confidence intervals, effect sizes, degrees of freedom and *P* value noted<br>*Give P values as exact values whenever suitable.*
☒	☐	For Bayesian analysis, information on the choice of priors and Markov chain Monte Carlo settings
☒	☐	For hierarchical and complex designs, identification of the appropriate level for tests and full reporting of outcomes
☒	☐	Estimates of effect sizes (e.g. Cohen's *d*, Pearson's *r*), indicating how they were calculated

Our web collection on statistics for biologists contains articles on many of the points above.

Software and code

Policy information about availability of computer code

Data collection	we don't have related issues.
Data analysis	we don't have related issues.

For manuscripts utilizing custom algorithms or software that are central to the research but not yet described in published literature, software must be made available to editors and reviewers. We strongly encourage code deposition in a community repository (e.g. GitHub). See the Nature Portfolio guidelines for submitting code & software for further information.

Data

Policy information about availability of data

All manuscripts must include a data availability statement. This statement should provide the following information, where applicable:

- Accession codes, unique identifiers, or web links for publicly available datasets
- A description of any restrictions on data availability
- For clinical datasets or third party data, please ensure that the statement adheres to our policy

We declare that all relevant data supporting the findings of this study are available within the article and its Supplementary Files or from the corresponding authors upon request.

Human research participants

Policy information about studies involving human research participants and Sex and Gender in Research.

Reporting on sex and gender	we don't have related issues.
Population characteristics	we don't have related issues.
Recruitment	we don't have related issues.
Ethics oversight	we don't have related issues.

Note that full information on the approval of the study protocol must also be provided in the manuscript.

Field-specific reporting

Please select the one below that is the best fit for your research. If you are not sure, read the appropriate sections before making your selection.

☒ Life sciences ☐ Behavioural & social sciences ☐ Ecological, evolutionary & environmental sciences

For a reference copy of the document with all sections, see nature.com/documents/nr-reporting-summary-flat.pdf

Life sciences study design

All studies must disclose on these points even when the disclosure is negative.

Sample size	In the characterization of circuit, usually two or three replicates are represented simultaneously, and the results of characterization and fitting have been shown in the manuscript. In fermentation experiment, error bars indicate three replicates.
Data exclusions	No data was excluded.
Replication	All experiments with explicit standard deviation (SD) were performed in triplicates.
Randomization	No data was randomized.
Blinding	For fluorescence observation and results analysis, the analytical team who analyze the data were blind to the samples that they were analyzing.

Reporting for specific materials, systems and methods

We require information from authors about some types of materials, experimental systems and methods used in many studies. Here, indicate whether each material, system or method listed is relevant to your study. If you are not sure if a list item applies to your research, read the appropriate section before selecting a response.

Materials & experimental systems

n/a	Involved in the study	
☒	☐	Antibodies
☒	☐	Eukaryotic cell lines
☒	☐	Palaeontology and archaeology
☒	☐	Animals and other organisms
☒	☐	Clinical data
☒	☐	Dual use research of concern

Methods

n/a	Involved in the study	
☒	☐	ChIP-seq
☐	☒	Flow cytometry
☒	☐	MRI-based neuroimaging

Flow Cytometry

Plots

Confirm that:

☒ The axis labels state the marker and fluorochrome used (e.g. CD4-FITC).

☒ The axis scales are clearly visible. Include numbers along axes only for bottom left plot of group (a 'group' is an analysis of identical markers).

☒ All plots are contour plots with outliers or pseudocolor plots.

☒ A numerical value for number of cells or percentage (with statistics) is provided.

Methodology

Sample preparation	Engineered E. coli were cultured in shake flasks containing 50 mL of LB medium at 200rpm, 30°C. Samples were taken at intervals and washed and diluted to OD less than 0.1 with phosphate buffered saline. The cytometry analysis was carried out by a flow cytometer (BD, USA). Fluorescence positive cells were captured under the excitation spectrum of 488nm (FITC channel, GFP), the channels of forward scatter (FSC) and side scatter (SSC).
Instrument	Flow Cytometer (BD FACSAria Fusion)
Software	Flow Jo
Cell population abundance	We only performed fluorescence analysis, not sorting.
Gating strategy	We set gate using FSC and SSC. The negative control group was E. coli MG1655. The positive control group was E. coli MG1655 expressing GFP.

☒ Tick this box to confirm that a figure exemplifying the gating strategy is provided in the Supplementary Information.
