# Supplementary material for: A synthetic population-level oscillator in non-microfluidic environments
Source: Commun Biol. 2023 May 13;6:515. doi: 10.1038/s42003-023-04904-0 (PMC10183009; doi:10.1038/s42003-023-04904-0)
Supplement: Supplementary file 1 — Liang_Peer Review File [file 42003_2023_4904_MOESM1_ESM.pdf]

nature portfolio

Peer Review File

A synthetic population-level oscillator in non-microfluidic environments.

Open Access This file is licensed under a Creative Commons Attribution 4.0 International License, which permits use, sharing, adaptation, distribution and reproduction in any medium or format, as long as you give appropriate credit to the original author(s) and the source, provide a link to the Creative Commons license, and indicate if changes were made. In the cases where the authors are anonymous, such as is the case for the reports of anonymous peer reviewers, author attribution should be to 'Anonymous Referee' followed by a clear attribution to the source work. The images or other third party material in this file are included in the article's Creative Commons license, unless indicated otherwise in a credit line to the material. If material is not included in the article's Creative Commons license and your intended use is not permitted by statutory regulation or exceeds the permitted use, you will need to obtain permission directly from the copyright holder. To view a copy of this license, visit http://creativecommons.org/licenses/by/4.0/.

Reviewers' comments:

Reviewer #1 (Remarks to the Author):

Comments for Communications Biology, "A synthetic population-level oscillator in non-microfluidic environments"

Overall Comments
The submitted manuscript from Gu et al. combines engineered quorum-sensing and protease-based feedback in an oscillation circuit which does not require external synchronization and would allow population control without microfluidics. The manuscript is generally well written, the presentation is clear. The topic is appropriate for the journal and the work adds to the body research in synthetic biology, specifically engineered oscillating circuits. The comments I have are relatively minor, and upon addressing these concerns, I recommend the manuscript be considered for publication.

Major Comments
• The work combines of the author's previously published Esa QS oscillating circuit and protease-activated degron regulation. While in the Introduction and Discussion much text is devoted to guiding the reader through oscillating circuits (which makes sense, as this is the main focus), the authors only briefly mention the protease-regulated degrons from Gao et al. The authors should add a few sentences to explain the development and importance of protease/degron negative feedback in synthetic circuit design. Furthermore, the Gao et al. work utilized previous work from Fernandez-Rodriguez and Voigt, which first demonstrated the use of the orthogonal TEV / TVMV / SUMMV proteases for regulated degradation by exposure of cryptic degron sites. See: Nucleic Acids Res. 2016 Jul 27; 44(13): 6493–6502. (doi: 10.1093/nar/gkw537). This paper should definitely be cited.
• One of the claims of novelty for this work is that it allows population synchronization without the use of microfluidics. While most of the literature indeed has used microfluidics to observe the dynamics of oscillating circuits, there is another case where population-level oscillation has been demonstrated in a non-microfluidic environment – the QS-based lysis circuit from Din and Danino et al. was demonstrate in an in vivo mouse model, where oscillations were detected in the mouse gut (see Figure 4 from Din, M., Danino, T., Prindle, A. et al. Synchronized cycles of bacterial lysis for in vivo delivery. Nature 536, 81–85 (2016).). This should be discussed.
• Figure 3d of the manuscript shows that AHL concentrations reach high levels during third oscillation (~ 30h), but the corresponding GFP reporter concentration for the third oscillation is poor (3b,c). Does this indicate circuit regulation breaking down? Does the larger third AHL peak indicate that AiiA is not degrading the AHL? Could the authors provide some insight into why the third AHL peak is much higher and sustained?
• Related to the above, for Figure 3, the amplitude differences of the GFP reporter (3b and 3c) seem to have the opposite trend as the detected AHL (3d), even though they are on the same positive feedback loop. That is, the first two cycles seem to have very high GFP and the third cycle has low GFP. In comparison, the AHL amounts for the first two cycles seems to be low, and the third cycle is high. Do the authors think this correlation is significant?
• Does Figure 3e really show "opposite oscillations," as the authors indicate (line 275)? There seems to be a slight phase shift, but the oscillations shown in 3e do not seem to have inverted phases.
• Line 299 - "There is also the potential to achieve more standardized and durable oscillations through circuit optimization in the future." From this study, what do the authors think are some key areas to improve in order to achieve more robust oscillators?
• For Figure 5, regulating cell morphology, there is no control set. There needs to be control data with the oscillating circuit without the FtsZ output, to make sure the changes in cell morphology are not due to metabolic stresses which may occur from other aspects of the circuit dynamics. For example, control cell length measurements from cells expressing only the QPK-H circuit, such as the one shown in Figure 4. It is known that cell morphology can change according to the growth phase. See: "Growth Phase-Coupled Alterations in Cell Structure and Function of Escherichia coli" J Bacteriol. 2003 Feb; 185(4): 1338–1345. (doi: 10.1128/JB.185.4.1338-1345.2003).
• Also, for Figure 5, there only seem to be two oscillations, based on the cell length metric, with the peaks occurring at 5 and 18h. In contrast, the same circuit using the GFP reporter (Figure 4)

shows three oscillations at 2, 9, and 30h. Could the authors discuss the difference here?
• For Figure 5d, what are the colors for the different violin plots?

Minor Comments
• The writing is good and concise, although it needs to be tightened up a bit, as there are quite a number of typos.
• Some typos, e.g., line 109: The oscillator is observed regular and robust population-level oscillations…
• Line 117: "…reemerge good oscillation…" unclear, needs clarification
• Line 182, typo.
• Line 196: By adjusting the direction of the proteolytic tag and the protease cleavage tag – what is meant here? Needs more clarification, could confuse the reader.

Reviewer #2 (Remarks to the Author):

In this paper, the authors took multiple steps to improve genetic oscillators to achieve multi-cycle oscillation in liquid culture conditions and showcased two examples of applying the genetic circuit to other scenarios. Inspired by previous work on synthetic biological oscillation designs, they first proposed designs with similar topologies but a new quorum-sensing (QS) system, the Esa system, and different protein degradation tags on the reporter GFP. Initially not able to achieve multiple oscillation cycles, the authors added additional proteinase regulation feedback structures coupled with the QS-based oscillation circuit. With the double-layered negative feedback design, they observed three-cycle oscillation in 24-well plates and shaking flasks. Next, they further improved the circuit's performance by reducing the metabolic burden by replacing the plasmid backbone and assembling the core circuit onto a single plasmid. At last, they individually coupled the E.coli morphology gene ftsZ and the MVA metabolism pathway with their circuit. They proved that the circuit has the potential to be applied to different conditions. It is impressive that the authors successfully improved the oscillator behavior to have multiple oscillations without use of delicate microfluidic devices. This truly illustrate the careful engineering and optimization needed to build robust dynamic behaviors. More importantly, this paper showed this is achievable, just not easy. The proposed strategy is insightful in synthetic genetic circuit designs. I would suggest its publication after addressing some of my concerns below.
Major concerns:
1. As mentioned in the paper, the oscillation behavior is obvious during the logarithm growth phase but becomes slow and lost after one cycle in the stationary phase. It appears that the oscillation behavior is tightly coupled with the growth process. As we know, AHLs can be freely diffused to the environment and other cells. Therefore, the AHL concentration is contributed by all cells in the culture. In other words, the AHL production and degradation are not only controlled by the genetic circuit in individual cells but also the cell density. The accumulation rate of AHLs is different at low cell density from that at high cell density. According to this paper, the AHL signal accumulation and resetting are the core factors of the emergence of oscillation. Given the fact that the oscillation can only last for one cycle at the stationary phase, I am curious to see some data/discussion on the possibility that the emergency of oscillation could be more effectively controlled by growth other than the genetic circuit.
2. (Line 110-112) The authors mentioned that their circuit design was inspired by a previous study using the Lux QS system. However, the topology appears the opposite. In that paper, the Plux promoter drives the expression of LuxI, which directly activates itself in a positive feedback manner. Then, the expression of aiiA, degrading the AHL can be regarded as long-term repression that resets the activation. The mechanism could be explained as fast and pulsive positive feedback expression succeeded by a delayed but strong negative feedback. In this study, however, the promoter PesaS has the opposite topology. The expression of esaI inhibits itself. Thus, the aiiA expression can be treated as an activation signal that removes the inhibition. So- what is the underlying mechanism of the emergency of oscillation? And, why did the authors choose to use the opposite topology? Does that cause any difference? These questions need to be addressed in the paper.

Minor concerns:

1. (Figure 1 and 2) The schematic graph showing the circuit design is misleading. According to the paper (Line 125-132), the AHL produced by EsaI can detach EsaR from the promoters PesaR or PesaS, to remove the repression or activation of the promoters, respectively. However, in the schematic graph, the information is that the EsaR-AHL complex directly activates or inhibits the promoters.
2. (Line 199-200) The phrase '… to achieve the stability and fast turnover of AiiA' should be 'to achieve the stable and fast turnover of AiiA'.

Response Letter to Reviewers

We appreciate the reviewers' comments and valuable suggestions. We have completed new analyses and revised the manuscript accordingly. We also performed additional experiments based on the suggestions. Our responses to the reviewers' comments are detailed below, and changes are highlighted in the revised manuscript.

Reviewer1

The submitted manuscript from Gu et al. combines engineered quorum-sensing and protease-based feedback in an oscillation circuit which does not require external synchronization and would allow population control without microfluidics. The manuscript is generally well written, the presentation is clear. The topic is appropriate for the journal and the work adds to the body research in synthetic biology, specifically engineered oscillating circuits. The comments I have are relatively minor, and upon addressing these concerns, I recommend the manuscript be considered for publication.

Response: Thank you very much for your time involved in reviewing the manuscript and your valuable comments. We are grateful for your help.

Major Comments

1. Comment: The work combines of the author's previously published Esa QS oscillating circuit and protease-activated degron regulation. While in the Introduction and Discussion much text is devoted to guiding the reader through oscillating circuits (which makes sense, as this is the main focus), the authors only briefly mention the protease-regulated degrons from Gao et al. The authors should add a few sentences to explain the development and importance of protease/degron negative feedback in synthetic circuit design. Furthermore, the Gao et al. work utilized previous work from Fernandez-Rodriguez and Voigt, which first demonstrated the use of the orthogonal TEV/TVMV/SUMMV proteases for regulated degradation by exposure of cryptic degron sites. See: Nucleic Acids Res. 2016 Jul 27; 44(13): 6493–6502. (doi: 10.1093/nar/gkw537). This paper should definitely be cited.

1. Response: Thank you for your suggestion, it's very helpful for the improvement of our manuscript. According to your suggestion, we have added relevant content to the manuscript to introduce the important role of protease regulatory elements in the design

of synthetic oscillators (Line 56-65), and cited the work of Fernandez-Rodriguez and Voigt, as well as other related work.

2. Comment: One of the claims of novelty for this work is that it allows population synchronization without the use of microfluidics. While most of the literature indeed has used microfluidics to observe the dynamics of oscillating circuits, there is another case where population-level oscillation has been demonstrated in a non-microfluidic environment – the QS-based lysis circuit from Din and Danino et al. was demonstrate in an in vivo mouse model, where oscillations were detected in the mouse gut (see Figure 4 from Din, M., Danino, T., Prindle, A. et al. Synchronized cycles of bacterial lysis for in vivo delivery. Nature 536, 81–85 (2016).). This should be discussed.

2. Response: Thank you for your suggestion. According to your suggestion, we have added relevant discussion and revised some expressions in the manuscript (Line 83-85, Line 97-102). Din and Danino et al. demonstrate the potential of synchronized lysis circuit as a drug delivery platform via co-culture with human cancer cells in vitro[1]. The synchronized lysis circuit enables the engineered bacteria to show "increase-decrease-increase" pulsatile population dynamics in vivo, demonstrating the potential of synthetic oscillators for biotherapy and drug delivery. It is worth further exploration to construct continuous population-level oscillators in non-microfluidic environment and explore its application potential in different fields.

3. Comment: Figure 3d of the manuscript shows that AHL concentrations reach high levels during third oscillation (~30h), but the corresponding GFP reporter concentration for the third oscillation is poor (3b,c). Does this indicate circuit regulation breaking down? Does the larger third AHL peak indicate that AiiA is not degrading the AHL? Could the authors provide some insight into why the third AHL peak is much higher and sustained?

3. Response: Thank you for your comment. It's a pleasure to discuss this issue with you and share some views. Although the fluorescence intensity of the third oscillation of the circuit decreased during the continuous culture, the periodic dilution characterization experiment showed that the circuit could still complete the oscillation behavior after dilution (Figure 2d). Moreover, flow cytometry analysis showed that a majority of

bacteria participated in the third oscillation (Figure 4b). Therefore, the circuit is in good working order. A recent study showed that the fraction of active ribosomes in *E. coli* reduced during periods of slow growth (including the stationary phase), thus affecting the rate of protein synthesis[2]. We speculate that the reason why the circuit cannot continue may be related to the reduced activity of the bacteria.

For the high and persistent peak of the third oscillation of AHL concentration, we believe the following reasons may be responsible. In this circuit, the expression of EsaR is constitutive, and more EsaR will accumulate after long-term culture. Therefore, it may take longer to accumulate enough AHL to initiate circuit at the population-level. In this process, although AHL synthase EsaI with degradation tag might have low expression level like GFP, AHL is relatively stable and accumulates gradually, so its third peak value is high.

For the low peak of the third oscillation of GFP, we believe that the following reasons may be responsible. The bacteria are in stationary phase, with relatively poor metabolic activity and large population size. Therefore, the expression of GFP with degradation tag may not be high, resulting in a lower peak after OD600 standardization.

4. Comment: Related to the above, for Figure 3, the amplitude differences of the GFP reporter (3b and 3c) seem to have the opposite trend as the detected AHL (3d), even though they are on the same positive feedback loop. That is, the first two cycles seem to have very high GFP and the third cycle has low GFP. In comparison, the AHL amounts for the first two cycles seems to be low, and the third cycle is high. Do the authors think this correlation is significant?

4. Response: Thank you for your comment. In our circuit, AHL synthase EsaI and GFP are regulated by the same promoter, so the variation trend of AHL and GFP should be consistent in theory. The results showed that the changes of oscillation period of the two are similar. The difference in amplitude between the two may be due to the following reasons. In this circuit, we regulate GFP expression using a strong degradation tag to demonstrate its transient changes, and the fluorescence characterization results are standardized by OD600. In the other hand, although EsaI with degradation tag might have low expression level like GFP, AHL is relatively stable

and accumulates gradually. So the difference in amplitude between the two is not a conflict.

This phenomenon may be explained more clearly combining with the results of flow cytometry analysis. The changes of AHL and GFP are correlated with the size of bacteria involved in the oscillation and the activity of bacteria at different growth stages. In the first two oscillation processes, the number of bacteria participating in the oscillation is small, and the metabolism of bacteria is more active. Therefore, the peak value of GFP oscillations after OD standardization is higher. The start-up time of the circuit is quick, meaning the short accumulation time of AHL, resulting in small amplitude of AHL. In the third oscillation, the bacteria are abundant and less active. Most of the bacteria participate in the oscillation and last for a long time. However, the bacteria migrate only at the weak fluorescence intensity, so the average fluorescence intensity is low. In this process, the start-up time of the circuit is longer, resulting in more accumulation of AHL.

5. Comment: Does Figure 3e really show “opposite oscillations,” as the authors indicate (line 275)? There seems to be a slight phase shift, but the oscillations shown in 3e do not seem to have inverted phases.

5. Response: Thank you for your comment. According to your reminding, we have revised the expression in the manuscript (Line 293-295).

6. Comment: Line 299 - “There is also the potential to achieve more standardized and durable oscillations through circuit optimization in the future.” From this study, what do the authors think are some key areas to improve in order to achieve more robust oscillators?

6. Response: Thank you for the comment. In view of this problem, we think the following several aspects might be helpful to optimize the circuit. First, we can reference the strategy of synchronizing the Repressilator, and optimize the circuit by analyzing error and reducing error propagation and information losses[3]. Second, we can optimize the circuit by precisely regulating the expression of key elements and by appropriate high-throughput screening methods. Recently, high-throughput screening strategies have made great progress in circuit optimization[4]. Third, the analysis of the

key topology of the oscillation circuit and the construction of a mathematical model may be helpful to further analyze and improve the characteristics of the circuit. In addition, inspired by your third comment, incorporating EsaR expression into the regulatory system may become more interesting in future design process. Moreover, it is helpful to design or optimize circuits by studying the structure of natural oscillators and applying synthetic elements with multiple levels regulation.

7. Comment: For Figure 5, regulating cell morphology, there is no control set. There needs to be control data with the oscillating circuit without the FtsZ output, to make sure the changes in cell morphology are not due to metabolic stresses which may occur from other aspects of the circuit dynamics. For example, control cell length measurements from cells expressing only the QPK-H circuit, such as the one shown in Figure 4. It is known that cell morphology can change according to the growth phase. See: "Growth Phase-Coupled Alterations in Cell Structure and Function of Escherichia coli" J Bacteriol. 2003 Feb; 185(4): 1338–1345. (doi: 10.1128/JB.185.4.1338-1345.2003).

7. Response: Thank you for your suggestion on improving the accessibility of our manuscript. According to your suggestion, we have performed the experiment of control set (Line 355-357, Supplementary Fig. 8). The relevant contents are provided below as a screen dump for your quick reference.

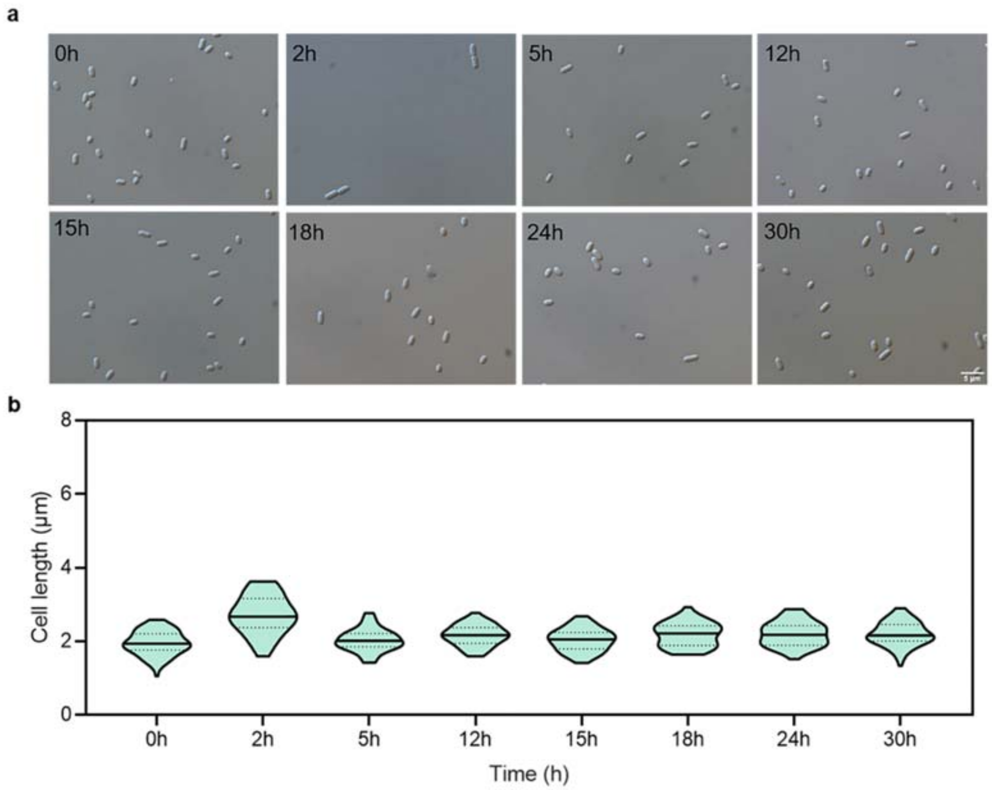

Supplementary Fig. 8. **a** Morphological observations of MG1655 expressing only the QPK-H circuit during culture. **b** Quantitative measurements of cell lengths by ImageJ from part a.

8. Comment: Also, for Figure 5, there only seem to be two oscillations, based on the cell length metric, with the peaks occurring at 5 and 18h. In contrast, the same circuit using the GFP reporter (Figure 4) shows three oscillations at 2, 9, and 30h. Could the authors discuss the difference here?

8. Response: Thank you for your comment. We believe that this may be related to time differences in transcriptional regulation, protein expression and assembly, and morphological transformation. GFP is quickly observed after expression. The FtsZ needs to be assembled into Z-ring to influence cell division and morphology. This may account for the difference in two characterization experiments.

9. Comment: For Figure 5d, what are the colors for the different violin plots?

9. Response: Thank you for your comment. The colors of the violin plots in the manuscript are for aesthetic purposes and have no special meaning. In order not to cause ambiguity, we have changed to a solid color display (Figure 5d).

Minor Comments

1. Comment: The writing is good and concise, although it needs to be tightened up a bit, as there are quite a number of typos.

1. Response: Thank you for the detailed review. We have carefully and thoroughly proofread the manuscript to correct the grammar and typos. We feel sorry for our carelessness.

2. Comment: Some typos, e.g., line 109: The oscillator is observed regular and robust population-level oscillations…

2. Response: Thank you for the detailed review. We have corrected it (Line 115-117).

3. Comment: Line 117: "…reemerge good

3. Response: Thank you for the detailed review. We have corrected it (Line 124-126).

We would like to take this opportunity to thank you for all your time involved and this great opportunity for us to improve the manuscript. We hope you will find this revised version satisfactory.

Sincerely,

The Authors

-----End of Reply to Reviewer #1------

Reviewer 2

In this paper, the authors took multiple steps to improve genetic oscillators to achieve multi-cycle oscillation in liquid culture conditions and showcased two examples of applying the genetic circuit to other scenarios. Inspired by previous work on synthetic biological oscillation designs, they first proposed designs with similar topologies but a new quorum-sensing (QS) system, the Esa system, and different protein degradation tags on the reporter GFP. Initially not able to achieve multiple oscillation cycles, the authors added additional proteinase regulation feedback structures coupled with the QS-based oscillation circuit. With the double-layered negative feedback design, they

observed three-cycle oscillation in 24-well plates and shaking flasks. Next, they further improved the circuit's performance by reducing the metabolic burden by replacing the plasmid backbone and assembling the core circuit onto a single plasmid. At last, they individually coupled the *E.coli* morphology gene *ftsZ* and the MVA metabolism pathway with their circuit. They proved that the circuit has the potential to be applied to different conditions. It is impressive that the authors successfully improved the oscillator behavior to have multiple oscillations without use of delicate microfluidic devices. This truly illustrate the careful engineering and optimization needed to build robust dynamic behaviors. More importantly, this paper showed this is achievable, just not easy. The proposed strategy is insightful in synthetic genetic circuit designs. I would suggest its publication after addressing some of my concerns below.

Response: Thank you very much for your time involved in reviewing the manuscript and your valuable comments. We are grateful for your help.

Major Comments

1. Comment: As mentioned in the paper, the oscillation behavior is obvious during the logarithm growth phase but becomes slow and lost after one cycle in the stationary phase. It appears that the oscillation behavior is tightly coupled with the growth process. As we know, AHLs can be freely diffused to the environment and other cells. Therefore, the AHL concentration is contributed by all cells in the culture. In other words, the AHL production and degradation are not only controlled by the genetic circuit in individual cells but also the cell density. The accumulation rate of AHLs is different at low cell density from that at high cell density. According to this paper, the AHL signal accumulation and resetting are the core factors of the emergence of oscillation. Given the fact that the oscillation can only last for one cycle at the stationary phase, I am curious to see some data/discussion on the possibility that the emergency of oscillation could be more effectively controlled by growth other than the genetic circuit.

1. Response: Thank you for your comment. Our experiments showed that the oscillations of QP-series circuits were weak and unstable during the stationary phase. We speculate that this condition is related to growth activity based on the following points. First, the characterization results showed that the circuit could still complete

oscillation behavior after successive dilution (Figure 2d). Flow cytometry analysis also showed that a majority of bacteria participated in the third oscillation process (Figure 4b). Therefore, we believe that the circuit is in good working order, and the weaker oscillation may not be very related to the breakdown of circuit. Second, in the repeated experiments of QP-M, the two oscillations that occurred during the log phase were stable, but the subsequent oscillations were more random, sometimes with two oscillations and sometimes with decreasing amplitude (Supplementary Fig. 4. The figure is shown below for easy viewing). The activated strains (such as retransforming plasmids into strain) could exhibit better oscillation behavior. Therefore, the growth status has some influence on the characterization of the circuit. Third, according to your comment, we performed an additional validation experiment to explore the relationship between bacterial growth status and gene expression. The expression of GFP was induced by adding IPTG at the early log phase, mid log phase and stationary phase, and the fluorescence intensity was measured after the same incubation time. The results showed that the intensity of GFP induced at the stationary phase was significantly reduced compared with the other two groups. Therefore, there is a certain correlation between growth status and gene expression (Supplementary Fig. 12. The figure is shown below for easy viewing). Fourth, a recent study showed that the fraction of active ribosomes in *E. coli* reduced during periods of slow growth (including the stationary phase), thus affecting the rate of protein synthesis[2]. Based on the above points, we believe that oscillation attenuation may be related to the growth status. Thank you again for your help in improving our work and we have added the relevant discussion to the manuscript (Line 242-244, Line 479-486).

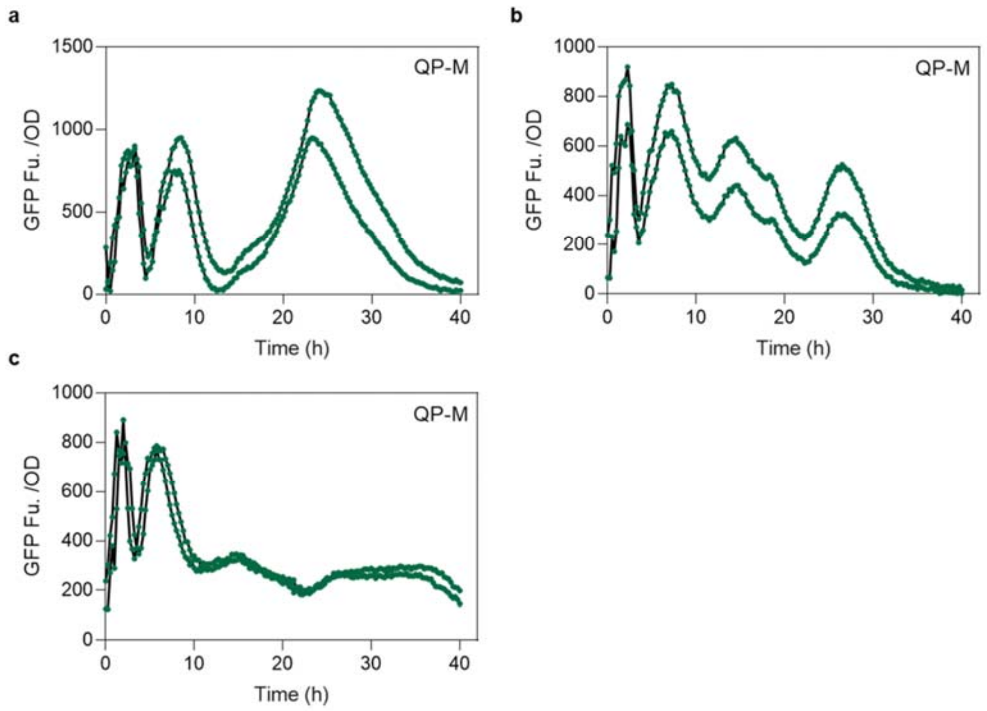

Supplementary Fig. 4. The repeated characterization of QP-M in 24-well plates. The circuit could maintain oscillation during the active growth period, that is, the first two oscillations were stable, but the subsequent oscillations might not be maintained.

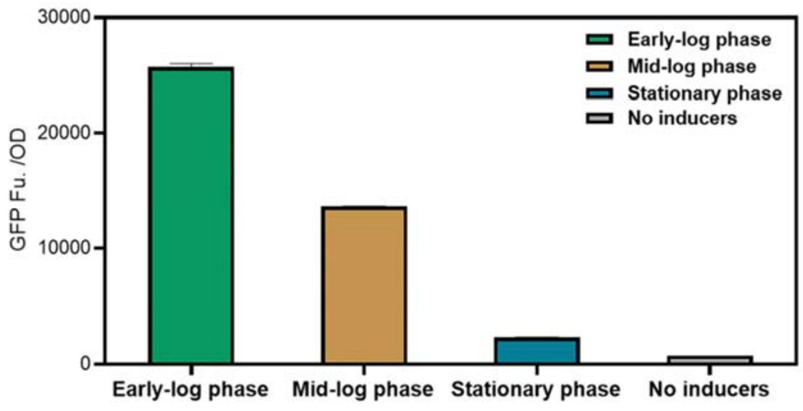

Supplementary Fig. 12. The fluorescence intensity of GFP, which was induced at the early log phase, mid log phase and stationary phase. The intensity of GFP induced at the stationary phase was reduced compared with the other two groups.

2. Comment: (Line 110-112) The authors mentioned that their circuit design was inspired by a previous study using the Lux QS system. However, the topology appears the opposite. In that paper, the P_{lux} promoter drives the expression of LuxI, which

directly activates itself in a positive feedback manner. Then, the expression of aiiA, degrading the AHL can be regarded as long-term repression that resets the activation. The mechanism could be explained as fast and pulsive positive feedback expression succeeded by a delayed but strong negative feedback. In this study, however, the promoter P_{esaS} has the opposite topology. The expression of esaI inhibits itself. Thus, the aiiA expression can be treated as an activation signal that removes the inhibition. So, what is the underlying mechanism of the emergency of oscillation? And, why did the authors choose to use the opposite topology? Does that cause any difference? These questions need to be addressed in the paper.

2. Response: Thank you for your comment on improving our manuscript. Our previous description may cause ambiguity, we have revised the relevant description (Line 120-122, Line 124-133). The original purpose of this work was to test the potential of synthetic oscillator in non-microfluidic environments. The synchronized genetic clock has been proved to be a promising population-level oscillator. We constructed the circuit based on the literature and tested it on a 24-well plate. The results showed that some efforts and attempts are still needed to make synthetic oscillators applicable in non-microfluidic environments. By analyzing the circuit composition, we speculated that the reason why the circuit could not oscillate continuously in the 24-well plate might be related to the signal reset in the large volume culture environment. The initiation of this circuit is caused by the leakage of LuxI to generate AHL. However, the leakage expression of LuxI with the degradation tag is relatively weak, and thus it is slow to restart the circuit in the stationary phase and on a large-scale culture. In addition, both the synthesis component LuxI and the degradation component AiiA are controlled by the same promoter P_{lux}, suggesting that the AHL synthesis and degradation processes are to some extent hedging, which may have an impact on the reset of QS signals under specific culture conditions. From the point of view of signal reset, we designed an new oscillator based on Esa QS-switch to separate the processes of signal synthesis and degradation.

Thank you again for your valuable comments. We have added the discussion of the underlying mechanisms of oscillation to the manuscript (Line152-159, Line 451-463).

In our circuit, EsaR activates the expression of EsaI and GFP, and inhibits the expression of AiiA. When AHL accumulates to a certain threshold, it binds to EsaR, thereby turning off the expression of EsaI and GFP, while turning on the expression of AiiA to degrade AHL. In this process, the accumulated AHL binds to EsaR* and turns off the expression of genes controlled by P_{esaS}, forming the delayed negative feedback at the population level to drive the emergence of oscillation. The mechanism is similar to the coupled Goodwin oscillator[5-10]. In synthetic oscillators, negative feedback prevents signal overload, which is necessary to carry a reaction network back to the "starting point" of its oscillation[11]. Signal reset is also crucial for coupling the oscillator to achieve continuous population-level oscillation, especially in large-scale environments. In the non-microfluidic environment, the accumulation of signal molecules will continue to increase, and the negative feedback at the transcriptional level may not be enough to complete signal reset. Therefore, we regulated the cascaded expression of AiiA to directly degrade signals to further prevent signal overload and accelerate signal reset. The activation element is designed as a measure to enhance the effect of negative feedback, similar to the effect of positive feedback in some oscillation circuits[12].

Minor concerns:

1. Comment: (Figure 1 and 2) The schematic graph showing the circuit design is misleading. According to the paper (Line 125-132), the AHL produced by EsaI can detach EsaR from the promoters P_{esaR} or P_{esaS}, to remove the repression or activation of the promoters, respectively. However, in the schematic graph, the information is that the EsaR-AHL complex directly activates or inhibits the promoters.

1. Response: Thank you for the detailed review. According to your suggestion, we have revised the schematic graphs in the manuscript (Figure 1a, Figure 1b, Figure 2a).

2. Comment: (Line 199-200) The phrase '… to achieve the stability and fast turnover of AiiA' should be 'to achieve the stable and fast turnover of AiiA'.

2. Response: Thank you for the detailed review. According to your suggestion, we have corrected the expression in the manuscript (Line 219-220).

We would like to take this opportunity to thank you for all your time involved and this great opportunity for us to improve the manuscript. We hope you will find this revised version satisfactory.

Sincerely,

The Authors

-----End of Reply to Reviewer #2------

References

1. Din, M.O. *et al.* Synchronized cycles of bacterial lysis for in vivo delivery. *Nature* **536**, 81-85 (2016).
2. Dai, X. *et al.* Reduction of translating ribosomes enables *Escherichia coli* to maintain elongation rates during slow growth. *Nat Microbiol* **2**, 16231 (2016).
3. Potvin-Trottier, L., Lord, N.D., Vinnicombe, G. & Paulsson, J. Synchronous long-term oscillations in a synthetic gene circuit. *Nature* **538**, 514-517 (2016).
4. Lezia, A., Csicsery, N. & Hasty, J. Design, mutate, screen: Multiplexed creation and arrayed screening of synchronized genetic clocks. *Cell Systems* **13**, 365-375 (2022).
5. Gonze, D. & Ruoff, P. The Goodwin Oscillator and its Legacy. *Acta Biotheoretica* **69**, 857-874 (2021).
6. Goodwin, B.C. Temporal Organization in Cells - a Dynamic Theory of Cellular Control Processes. *Current Science* **34**, 648-& (1965).
7. Goodwin, B.C. Oscillatory behavior in enzymatic control processes. *Adv Enzyme Regul* **3**, 425-438 (1965).
8. Komin, N., Murza, A.C., Hernandez-Garcia, E. & Toral, R. Synchronization and entrainment of coupled circadian oscillators. *Interface Focus* **1**, 167-176 (2011).
9. Fraser, A. & Tiwari, J. Genetical feedback-repression. II. Cyclic genetic systems. *J Theor Biol* **47**, 397-412 (1974).
10. Ruoff, P. & Rensing, L. The temperature-compensated goodwin model simulates many circadian clock properties. *Journal of Theoretical Biology* **179**, 275-285 (1996).
11. Novak, B. & Tyson, J.J. Design principles of biochemical oscillators. *Nature Reviews Molecular Cell Biology* **9**, 981-991 (2008).
12. Ananthasubramaniam, B. & Herzel, H. Positive feedback promotes oscillations in negative feedback loops. *PLoS One* **9**, e104761 (2014).

REVIEWERS' COMMENTS:

Reviewer #1 (Remarks to the Author):

The changes to the revised manuscript by the authors have adequately addressed the concerns raised during review. I recommend the revised manuscript for publication.

Reviewer #2 (Remarks to the Author):

No more comments. All looks good to me.

Response Letter to Reviewers

We appreciate the reviewers' approval and help.

Reviewer1

The changes to the revised manuscript by the authors have adequately addressed the concerns raised during review. I recommend the revised manuscript for publication.

Response: Thank you very much for your time involved in reviewing the manuscript and your positive comments. We are grateful for your help in improving the manuscript.

Reviewer2

No more comments. All looks good to me.

Response: Thank you very much for your time involved in reviewing the manuscript and your positive comments. We are grateful for your help in improving the manuscript..

Sincerely,

Quanfeng Liang and Qingsheng Qi

State Key Laboratory of Microbial Technology, Shandong University, No.72 Binhai Road, Qingdao, 266237, People's Republic of China

Email: liangquanfeng@sdu.edu.cn

qiqingsheng@sdu.edu.cn
